# Prevalence and transmission risk of colistin and multidrug resistance in long-distance coastal aquaculture

**DOI:** 10.1038/s43705-023-00321-w

**Published:** 2023-11-07

**Authors:** Taicheng An, Yiwei Cai, Guiying Li, Shaoting Li, Po Keung Wong, Jianhua Guo, Huijun Zhao

**Affiliations:** 1 Guangdong-Hong Kong-Macao Joint Laboratory for Contaminants Exposure and Health, Guangdong Key Laboratory of Environmental Catalysis and Health Risk Control, Institute of Environmental Health and Pollution Control, Guangdong University of Technology, Guangzhou, 510006 China; 2 Guangzhou Key Laboratory of Environmental Catalysis and Pollution Control, Key Laboratory of City Cluster Environmental Safety and Green Development of the Ministry of Education, School of Environmental Science and Engineering, Guangdong University of Technology, Guangzhou, 510006 China; 3 College of Biological and Pharmaceutical Science, Guangdong University of Technology, Guangzhou, 510006 China; 4 Australian Centre for Water and Environmental Biotechnology, The University of Queensland, St. Lucia, Brisbane, QLD 4072 Australia; 5 Centre for Clean Environment and Energy, and Griffith School of Environment, Gold Coast Campus, Griffith University, Gold Coast, QLD 4222 Australia

**Keywords:** Antibiotics, Microbial ecology

## Abstract

Due to the wide use of antibiotics, intensive aquaculture farms have been recognized as a significant reservoir of antibiotic resistomes. Although the prevalence of colistin resistance genes and multidrug-resistant bacteria (MDRB) has been documented, empirical evidence for the transmission of colistin and multidrug resistance between bacterial communities in aquaculture farms through horizontal gene transfer (HGT) is lacking. Here, we report the prevalence and transmission risk of colistin and multidrug resistance in 27 aquaculture water samples from 9 aquaculture zones from over 5000 km of subtropical coastlines in southern China. The colistin resistance gene *mcr−1*, mobile genetic element (MGE) *intl1* and 13 typical antibiotic resistance genes (ARGs) were prevalent in all the aquaculture water samples. Most types of antibiotic (especially colistin) resistance are transmissible in bacterial communities based on evidence from laboratory conjugation and transformation experiments. Diverse MDRB were detected in most of the aquaculture water samples, and a strain with high-level colistin resistance, named *Ralstonia pickettii* MCR, was isolated. The risk of horizontal transfer of the colistin resistance of *R. pickettii* MCR through conjugation and transformation was low, but the colistin resistance could be steadily transmitted to offspring through vertical transfer. The findings have important implications for the future regulation of antibiotic use in aquaculture farms globally to address the growing threat posed by antibiotic resistance to human health.

## Introduction

Antibiotic resistance has become a major threat to public health worldwide, directly causing more than 1.27 million deaths in 2019 alone [1]. As antibiotic resistance can spread through horizontal gene transfer (HGT), the emergence and transmission of multidrug-resistant bacteria (MDRB) poses a serious burden to public health [2, 3]. Due to the lack of effective and biocompatible drugs, infections caused by multidrug-resistant Gram-negative bacteria have been listed as one of the most urgent global health threats [4].

In addition to the use of antibiotics in clinical settings, various antibiotics have been intensively used in livestock, poultry, and aquaculture farms, thus promoting the acquisition of antibiotic resistance [5, 6]. The amount of antibiotics used in the feed of livestock, poultry, and aquatic animals, such as fish and shrimp, is growing rapidly and is already twice the amount prescribed to humans each year [7]. As a consequence, livestock, poultry, and aquaculture ecosystems have been identified as hotspots harboring diverse antibiotic resistomes, including antibiotic resistance genes (ARGs), antibiotic-resistant bacteria (ARB), and even MDRB. Antibiotic resistance in terrestrial and aquatic food animals has also become an emerging concern. For example, *Salmonella spp*. and *Campylobacter spp*., the two most common zoonotic pathogens causing foodborne illness in the UK, were isolated from human and animal sources and showed overall similar antimicrobial resistance [8]. In addition, multidrug-resistant *Edwardsiella tarda*, a common fish pathogen, has not only been isolated from the aquatic animals Nile tilapia and African catfish [9] but also been shown to cause disease in humans [10]. In addition, abundant ARB and ARGs may accelerate the transmission risk of the emergence and transmission of MDRB in livestock, poultry and aquaculture environments [11–13]. For example, colistin is commonly used to treat MDRB infections, and it was first used to treat Gram-negative bacterial infections over 50 years ago; its use has since gradually reduced due to its severe impairment of renal function, and it is reserved as a last-line of antibiotic [14]. The use of colistin in Vietnam has been documented [15, 16], while the use of colistin in aquaculture in China is rarely reported. Nevertheless, *mcr−1* was highly prevalent in *Escherichia coli* strains isolated from meat and chicken in slaughterhouses and supermarkets in four Chinese provinces [17], suggesting that *mcr−1* is widespread in livestock and poultry farms.

It was hypothesized that the intensive use of antibiotics in aquaculture could impose selection pressure on microorganisms, thus likely accelerating the transmission risk of *mcr−1* among microbes through HGT [18, 19], which includes conjugation, transformation and transduction. Specifically, conjugation is a process by which one donor cell transfers ARGs to a recipient through direct cell contact, while transduction is mediated by bacteriophages to transfer ARGs among bacteria. Compared to conjugation and transduction, the process of transformation does not require a living donor cell but needs only the presence of persistent extracellular ARGs [11–13]. Although antibiotic resistomes (MDRB, ARB, and ARGs) have been widely detected [20, 21], there is a lack of empirical evidence for HGT among bacterial communities in aquaculture farms.

The objective of this study was to investigate the prevalence and transmission risk of colistin and multidrug resistance in long-distance coastal aquaculture. To this end, we employed both culture-dependent and culture-independent methods to analyze the diversity profiles of typical antibiotic resistomes with particular attention to colistin resistance, from over 5000 km in subtropical coastal aquaculture environments in southern China. More importantly, we established both conjugation and transformation models to assess the transmission risk of various ARGs in samples, with emphasis on the colistin resistance gene *mcr−1*. In addition, an MDRB strain with high levels of colistin resistance in water samples was isolated and characterized, including by whole-genome sequencing, multidrug resistance profiling, and antibiotic resistance transmission risk assessment. Collectively, the findings provide insights into the transmission of colistin resistance/multidrug resistance in aquaculture environments.

## Methods and materials

### Water sample collection

The subtropical coastal cities in South China are the main bases for aquaculture of seafood, producing a large amount of seafood and supplying it to the world every year. From March to June 2021, a total of nine subtropical offshore mixed aquaculture water samples were collected from seven aquaculture ponds together with two clean water samples of the control sites in the aquaculture area with over 5000 km of coastline in the subtropical coastal waters of southern China. Detailed information on the designated sites for water sampling is shown in Fig. S1 (Supplementary Information, SI). The water samples were named GX, FC, QZ, GD, ZJ1, ZJ2, YJ, ZH, and HZ according to the sampling cities, among which GX and GD were the control samples. Three replicate samples (50−80 m intervals) were collected at each sampling point using a 2 L sterile water sampler. In total, 27 samples were collected from these sites. After mixing replicated samples from each site, 300 mL of water was immediately filtered through a 0.22 μm sterile polycarbonate membrane (Millipore Corporation, USA). The filters were immediately frozen in liquid nitrogen and subsequently stored at -80 °C for subsequent DNA extraction. The details of DNA extraction and qPCR analysis are shown in Text S1. In addition, 100 mL water from mixed samples from each site was stored at 4 °C for subsequent strain selection, HGT, and community diversity analysis.

### Conjugative transfer assay of indigenous bacterial populations

To explore whether bacterial antibiotic resistance can spread among indigenous bacterial populations, conjugative transfer was directly assessed in each aquaculture water sample. Given that indigenous bacterial populations are more representative of real conjugative transfer efficiency, this study employed *E. coli* C600 (for which the minimal inhibitory concentration (MIC) of streptomycin was 4096 mg L^−1^) as the recipient, while bacterial populations with different antibiotic resistance in aquaculture water were used as the donors. The bacterial populations in aquaculture water were concentrated and adjusted to 10^8^ CFU mL^−1^, and the same volume of donor and recipient (10^8^ CFU mL^−1^) were mixed evenly in phosphate-buffered saline (PBS). Conjugation mating systems were treated for 12 h at 37 °C without shaking. Another recipient was taken from the medium alone as the control, and treated for 12 h at 37 °C without shaking. The cultured mixed bacterial suspension was diluted with a tenfold gradient in PBS, and 100 μL of the dilution was spread on a MacConkey agar plate (as a selective medium on which the growth of Gram-positive bacteria was inhibited), which was supplemented with an appropriate amount of different target antibiotics (2 mg L^−1^ cefotaxime, 8 mg L^−1^ meropenem, 2 mg L^−1^ colistin, 8 mg L^−1^ chloramphenicol, 2 mg L^−1^ kanamycin, 1 mg L^−1^ vancomycin, 1 mg L^−1^ lincomycin, 1 mg L^−1^ tetracycline, 1 mg L^−1^ rifampicin and 1 mg L^−1^ sulfisoxazole) and 3000 mg L^−1^ streptomycin; only the *E. coli* transconjugants grew on this medium as pink colonies on. In total, nine mating experiments were performed on water samples from all sites. The conjugative spread efficiency was calculated as the number of transconjugants obtained per recipient, following a method described previously [22], as follows:

$${{{{{\rm{Conjugative}}}}}}\; {{{{{\rm{spread}}}}}}\; {{{{{\rm{efficiency}}}}}}={{{{{\rm{Transconjugants}}}}}}({{{{{\rm{CFU}}}}}}\,{{{{{{\rm{mL}}}}}}}^{-1})/{{{{{\rm{Recipients}}}}}}({{{{{\rm{CFU}}}}}}\,{{{{{{\rm{mL}}}}}}}^{-1})$$

### Plasmid transformation assay

Detailed procedures for plasmid extraction from indigenous bacterial populations are given in Text S2. Competent *E. coli* DH5α (Sangon Biotech, China) was used as the recipient, and the extracted bacterial plasmids from each aquaculture water sample were used as the target DNA for transformation. Competent *E. coli* DH5α (100 μL) cells were taken from −80 °C, immediately placed on ice, and held there for 5 − 10 min. Then, 10 μL of target DNA was added, and the sample was shaken gently and placed on ice for 20 min. After being gently shaken again, the sample was inserted into a water bath at 42 °C for 1−2 min for heat shock, then quickly put back on ice and allowed to stand for 3−5 min. Approximately 500 μL of LB medium was added to each of the above tubes, followed by shaking at 37 °C for 1 h. The sample was spread on a nutrient agar (NA) with appropriate amounts of different target antibiotics (2 mg L^−1^ cefotaxime, 8 mg L^−1^ meropenem, 2 mg L^−1^ colistin, 8 mg L^−1^ chloramphenicol, 2 mg L^−1^ kanamycin, 1 mg L^−1^ vancomycin, 1 mg L^−1^ lincomycin, 1 mg L^−1^ tetracycline, 1 mg L^−1^ rifampicin and 1 mg L^−1^ sulfisoxazole) and incubated overnight, and then the number of colonies grown was recorded. The transformative spread efficiency was calculated as the amount of target DNA obtained per recipient as follows:

$${{{{{\rm{Transformative}}}}}}\; {{{{{\rm{spread}}}}}}\; {{{{{\rm{efficiency}}}}}}={{{{{\rm{Transformants}}}}}}({{{{{\rm{CFU}}}}}}\,{{{{{{\rm{mL}}}}}}}^{-1})/{{{{{\rm{Recipients}}}}}}({{{{{\rm{CFU}}}}}}\,{{{{{{\rm{mL}}}}}}}^{-1})$$

### Selection and abundance determination of MDRB

Because the MICs of some antibiotics (including vancomycin, lincomycin, tetracycline, rifampicin, erythrocin, ofloxacin, and sulfisoxazole) for the quality control strain was too low (<1 mg L^−1^) (Table S1), accurate concentrations could not be used for culturable MDRB selection. Therefore, cefotaxime, meropenem, colistin, chloramphenicol and kanamycin were selected as antibiotics for culturable MDRB selection. Cefotaxime (2 mg L^−1^), meropenem (8 mg L^−1^), colistin (2 mg L^−1^), chloramphenicol (8 mg L^−1^), and kanamycin (2 mg L^−1^) were added to sterilized NA and marine bacterial medium (2216 agar), prepared as 20 mL of solid medium per dish. The collected water samples were diluted with inactivated artificial seawater at appropriate gradients and 1 mL was spread on NA and 2216 Agar to select culturable MDRB that could survive in freshwater and seawater environments, respectively. Considering that the freshwater environment may have an impact on human domestic water, it is very important to determine whether MDRB can survive and proliferate in the freshwater environment.

### Selection, identification, and whole-genome sequencing of the high-level colistin-resistant strain

Selection was performed again using freshwater medium and seawater medium with a high level of colistin (200 mg L^−1^) from the selected MDRB. Bacterial 16S rRNA Sanger sequencing was performed using the 27F and 1492R pair. Detailed procedures for identification and whole-genome sequencing are described in Text S4 and S5.

### Multidrug resistance profile assay of the high-level colistin-resistant strain

Multidrug resistance profiles of *Ralstonia pickettii* MCR (*R. pickettii* MCR), including the resistance to cefotaxime, imipenem, meropenem, kanamycin, colistin, vancomycin, lincomycin, tetracycline, chloramphenicol, erythrocin, rifampicin, sulfisoxazole and ofloxacin, were assayed according to the quantitative analysis results of ARGs in water samples. The interpretive criteria for MICs were based on the European Committee on Antibiotic Susceptibility Testing (EUCAST, Version 13.1, www.eucast.org). *R. pickettii* MCR grown to log phase was prepared as a bacterial suspension at OD_600nm_ = 0.1, and then diluted 100-fold with nutrient broth (to ~10^6^ CFU mL^−1^). The antibiotics were incorporated into 96-well plates containing nutrient broth medium in serial twofold dilutions, followed by the addition of an equal amount of the diluted bacterial suspension and incubation at 37 °C overnight. Antibiotic resistance was assessed by identifying wells containing MICs that inhibited its growth.

### Other assays

Detailed protocols for “DNA extraction and qPCR analysis”, “plasmid extraction”, “MDRB diversity analysis” and other assays are shown in SI.

## Results

### Diverse ARGs and MDRB were detected in over 5000 km of aquaculture water

**Fig. 1:**
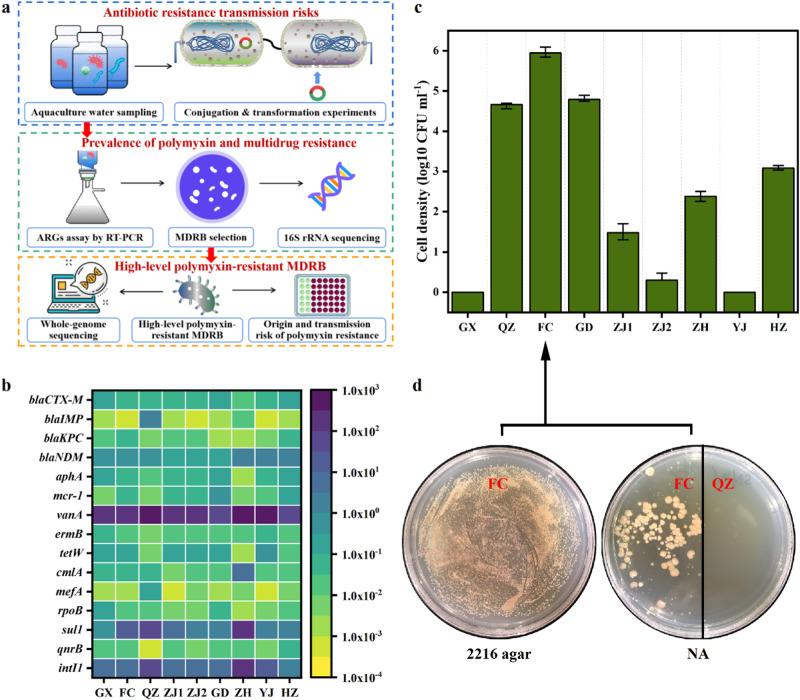
Diverse ARGs and MDRB detected in over 5000 km aquaculture waters. **a** Schematic of the experimental design. **b** Various ARGs profiles in long-distance aquaculture water samples. **c** Total culturable MDRB density (resistant to cefotaxime (2 mg L^−1^), meropenem (8 mg L^−1^), colistin (2 mg L^−1^), chloramphenicol (8 mg L^−1^), and kanamycin (2 mg L^−1^)), error bars: standard deviation of colony-forming units (CFU) of culturable bacterial cells. **d** Selection of culturable MDRB in seawater medium (2216 agar) and freshwater medium (NA). FC: water sample FC; QZ: water sample QZ.

ARGs in the environment can be captured by pathogenic bacteria through the HGT process to form new ARB, even MDRB. In this study, antibiotic resistomes profiles were systematically investigated in long-distance aquaculture water samples (Fig. 1a). First, this study determined the relative abundance of ARGs in the collected samples (Fig. 1b). The ARGs *vanA* and *sul1* exhibited high abundance in the samples (7.75 × 10−9.09 × 10^2^/16S rRNA and 9.1 × 10^−1^−8.22 × 10/16S rRNA), and their abundance was highest in QZ and ZH. These were followed by *blaCTX-M*, *blaNDM*, *aphA*, *mcr−1*, *ermB*, *tetW*, *cmlA*, *rpoB*, and *qnrB*, which were moderately abundant in the collected samples. Among these ARGs, *mcr−1* was also detected at moderate levels (4.12 × 10^−3^−4.37 × 10^−1^/16S rRNA) in all samples, suggesting that it was widely prevalent in the aquaculture environment. Furthermore, high levels of the mobile genetic element (MGE) *intI1* were detected in the all samples (Fig. 1b), suggesting that the ARGs in these waters likely have a high transfer risk.

The abundance of MDRB can clearly reflect the transmission risk of antibiotic resistance. The total culturable MDRB density varied among different samples (Fig. 1c). The presence of culturable MDRB was undetectable in YJ and GX, while varying levels (10−10^6^ CFU mL^−1^) were detected in the other samples. Among them, the highest culturable MDRB abundance (~10^6^ CFU mL^−1^) was detected in FC. We further used a common bacterial proliferation medium to measure the abundances of culturable MDRB. As shown in Fig. 1d, most of the culturable MDRB in marine culture medium could not grow on the common medium, except for FC.

**Fig. 2:**
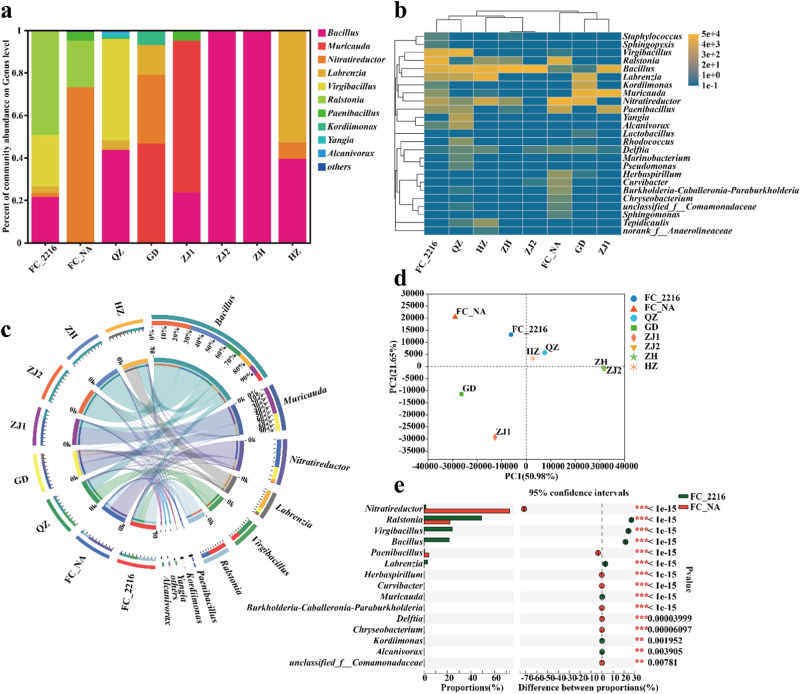
Microbial community analysis of MDRB. **a** Abundance distribution of each sample at genus level of MDRB. **b** Heat map of microbial community structure at the genus level. **c** Visualization circle map of the top 10 dominant genera. **d** The PCA analysis at genus level. **e** Significance analysis of genera exhibiting abundance differences in FC_2216 and FC_NA microbial communities.

This study further revealed the microbial communities of MDRB based on culturable MDRB colony 16S rRNA gene amplicon sequencing (Fig. 2). At the genus level, *Bacillus*, *Muricauda* and *Nitratireductor* were dominant in most of the samples. Bacteria from the FC sample propagated in seawater medium and normal medium exhibited different composition profiles, but a higher abundance of *Ralstonia* was observed in both media. The community diversity heatmap showed similar dominant bacterial genera, further supporting the above results (Fig. 2b). The distribution of dominant genera in each sample was also visualized with a circle map (Fig. 2c). *Bacillus*, *Muricauda*, *Nitratireductor*, *Labrenzia*, *Virgibacillus* and *Ralstonia* dominated in all the samples. *Ralstonia* was mainly distributed in the samples FC_2216 and FC_NA and could proliferate in both seawater and normal medium, suggesting that *Ralstonia* is a key genus that adapts to seawater and freshwater environments. To further compare community similarities among all samples, principal component analysis (PCA) of community diversity was performed (Fig. 2d). FC_2216 and FC_NA, HZ and QZ, ZH and ZJ2, and GD and ZJ1 showed high similarity. Although the proportion of *Ralstonia* in the two samples differed by nearly 30%, it was the only genus that could proliferate well in both media, proving that *Ralstonia* may survive in both seawater and freshwater (Fig. 2e).

In summary, typical ARGs are widely distributed in this aquaculture environment, and even the colistin resistance gene *mcr−1* is widespread. In addition, based on microbial community analysis, the diversity of culturable MDRB in water samples varied. *Ralstonia* may proliferate in both seawater and freshwater media.

### ARG transmission risks via conjugation and transformation

**Fig. 3:**
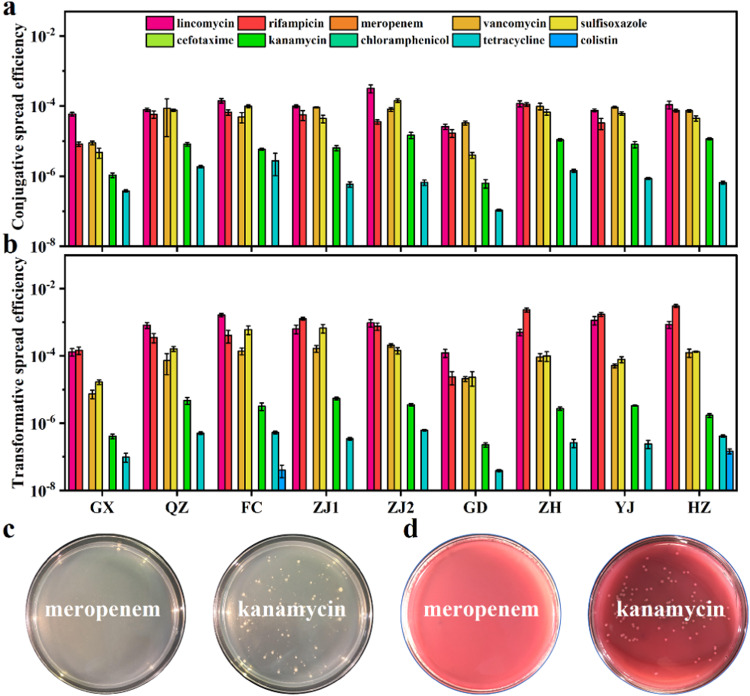
Horizontal transfer experiments of antibiotic resistance in aquaculture waters. **a** Indigenous bacterial populations conjugative transfer assay. **b** Plasmid DNA-mediated transformation experiments. **c** Transformants selected by transformation experiments. **d** Transconjugants selected by conjugation experiments using MacConkey agar.

This study further established both conjugation and transformation models to assess the transfer risk of antibiotic resistance in aquaculture waters. First, this study determined the conjugative spread efficiency of antibiotic resistance in water samples. As shown in Fig. 3a, not all antibiotic resistance was transferred to the recipient through conjugation, and the conjugative spread efficiencies of different types of antibiotic resistances differed widely. Among them, the conjugative spread efficiency of tetracycline resistance was low (10^−7^−10^−5^), while the conjugative spread efficiency of kanamycin resistance was moderate (10^−6^−10^−4^). The conjugative spread efficiencies of rifampicin, vancomycin, and sulfisoxazole resistance were all high (10^−6^−10^−3^), while that of lincomycin was the highest (10^−5^−10^−3^). Notably, no conjugative spread of meropenem, cefotaxime, chloramphenicol, or colistin resistance occurred. The above results suggest that some types of antibiotic resistance cannot be transferred to recipients through plasmid-mediated conjugation. In addition, the diverse conjugative spread efficiencies of different ARGs were likely related to their abundance in aquaculture environments and the fitness cost of plasmids [23].

In plasmid-mediated transformation (Fig. 3b), tetracycline resistance spread less efficiently (10^−7^−10^−6^). Colistin resistance was transformed only in FC and HZ with spread efficiencies of 0.4 × 10^−7^ and 1.47 × 10^−7^, respectively. Rifampicin, lincomycin, vancomycin, and sulfisoxazole resistance occurred with higher transformative spread efficiencies (10^−6^−10^−3^). Colonies of different sizes were observed on agar with antibiotics applied to select transformants or transconjugants (Fig. 3c, d), suggesting that the fitness cost for each bacterium within the recipient colony was not the same. Therefore, meropenem, cefotaxime, and chloramphenicol resistance cannot spread through conjugation or transformation. On the contrary, rifampicin, lincomycin, vancomycin, and sulfisoxazole resistances could spread via conjugation or transformation with high efficiency.

### The colistin-resistant strain in aquaculture water

**Fig. 4:**
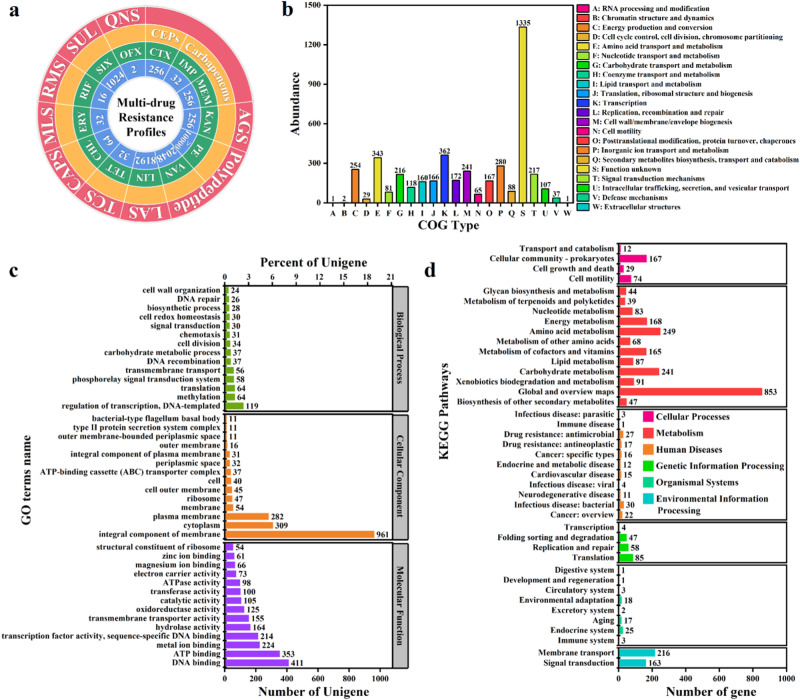
Multidrug resistance and genome annotation of the high-level colistin-resistant *Ralstonia pickettii* MCR. **a** Multidrug resistance profile. AGS aminoglycosides, LAS lincosamides, TCS tetracyclines, CAPS chloramphenicols, MLS macrolides, RMS rifamycins, SUL sulfonamides, QNS quinolones, CEPs cephalosporins, CTX cefotaxime, IMP imipenem, MEM meropenem, KAN kanamycin, PE colistin, VAN vancomycin, LIN lincomycin, TET tetracycline, CHL chloramphenicol, ERY erythrocin, RIF rifampicin, SIX sulfisoxazole, OFX ofloxacin. **b** COG database annotation. **c** GO database annotation. **d** KEGG database annotation.

There was an unknown strain in the FC sample that could proliferate on both seawater and freshwater media. By further selection in medium with a high level of colistin (200 mg L^−1^), a high-level colistin-resistant MDRB was obtained. This strain was identified as *Ralstonia pickettii* based on BLAST similarity and was named *R. pickettii* MCR. *R. pickettii* MCR exhibited resistance to 13 antibiotics (Fig. 4a). Among them, the resistance to ofloxacin was low (2 mg L^−1^); the resistance to five antibiotics (imipenem, tetracycline, chloramphenicol, erythrocin and rifampicin) was moderate (16−64 mg L^−1^); the resistance to cefotaxime, meropenem and kanamycin was moderate to high (256 mg L^−1^); the resistance to sulfisoxazole, lincomycin, and vancomycin was high (1024–8192 mg L^−1^); and the resistance to colistin was extremely high (>10,000 mg L^−1^). Thus, *R. pickettii* MCR carried at least five types of antibiotic resistance, of which colistin resistance reached high levels.

To investigate the origin and transfer risk of *R. pickettii* MCR colistin resistance, we performed a genomic analysis. First, we annotated the predicted coding genes with three major databases (COG, GO and KEGG) (Fig. 4b–d). In terms of the COG annotations (Fig. 4b), genes involved in defense mechanisms may be the main origin of the multidrug resistance and high-level colistin resistance of *R. pickettii* MCR. Their COG descriptions mainly include ATP-binding cassette (ABC) transporter, ABC-2 type transporter, iron ion homeostasis, confers resistance to bacitracin, efflux transporter RND family, mate efflux family protein, secretion protein HlyD family, beta-lactamase and restriction endonuclease. In terms of the GO annotations (Fig. 4c), 56 genes involved in transmembrane transport in the biological process may be the main source of multidrug resistance in *R. pickettii* MCR. Twenty-six genes involved in DNA repair may contribute to the recovery of *R. pickettii* MCR from antibiotic treatment, leading to antibiotic resistance. In addition, genes involved in the response to oxidative stress and the SOS response may also contribute to the response of the strain to different external stress conditions. Among the major cellular components, 37 genes involved in the ABC transporter complex may contribute to the efflux of multiple drugs. Several genes involved in membrane components encode multidrug efflux-related functions, such as the ABC transporter superfamily, mate efflux family, and RND efflux family genes. Among the major molecular functions, many genes involved in transmembrane transporter activity and transferase activity may contribute to the efflux of multiple drugs. In addition, genes involved in ATP binding and ATPase activity ensure energy supply during efflux. The above functions are likely to contribute to the multidrug resistance and high-level colistin resistance of *R. pickettii* MCR. In terms of the KEGG annotations (Fig. 4d), genes involved in transport and catabolism in cellular processes and membrane transport in environmental information processing play important roles in multidrug efflux. Energy metabolism provides a guarantee for multidrug efflux. In addition, genes involved in drug resistance: antimicrobial in human diseases and environmental adaptation in organismal systems may be the main origin of the multidrug resistance and high-level colistin resistance of *R. pickettii* MCR. Some genes implicated in human diseases, such as those involved in infectious disease: bacterial and immune disease, may indicate the potential pathogenicity of *R. pickettii* MCR. Therefore, the genome of *R. pickettii* MCR encoded many genes related to efflux and bacterial resistance to stress, resulting in its multidrug resistance, including colistin resistance.

**Fig. 5:**
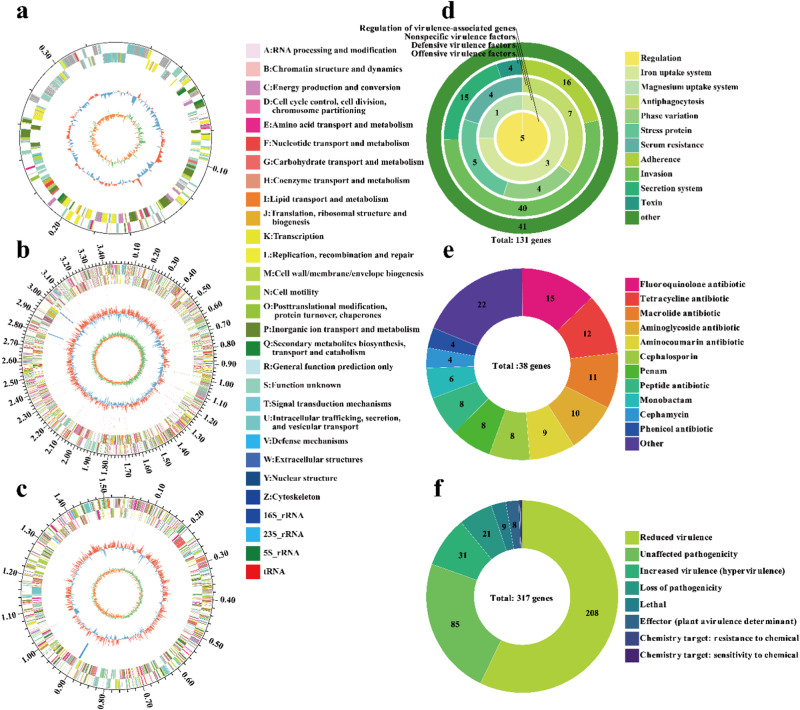
Genome circos maps and pathogenicity analysis of *Ralstonia pickettii* MCR. **a** COG annotation of plasmid. **b** COG annotation of chromosome 1. **c** COG annotation of chromosome 2. **d** Predictive analysis of virulence genes. **e** Predictive analysis of ARGs. **f** Predictive analysis of genes related to pathogen and host interaction.

The sequences of two chromosomes and one plasmid of *R. pickettii* MCR were assembled. The lengths of the two chromosomes were 3,486,479 bp and 1,533,404 bp, respectively, while the length of the plasmid was 336,372 bp. Subsequently, the genome circos map of the plasmid (Fig. 5a), chromosome 1 (Fig. 5b) and chromosome 2 (Fig. 5c) of *R. pickettii* MCR were illustrated. The genome circos map was the result of COG functional annotation of each coding gene. Among all the annotated functions of the plasmid pRp12D02 (named by NCBI), five genes encoded conjugation transfer-related proteins, including the conjugation transfer proteins Trbi and TrbG as well as type IV secretion system protein. Two genes were integrase family genes, and nine genes encoded transposase. Five genes encoded the phage integrase family protein. Two genes encoded the efflux transporter RND family, and 11 genes encoded metal resistance, which may lead to multidrug resistance and the resistance of *R. pickettii* MCR to external stress. In addition, 10 potential conjugation-related genes and 14 potential ARGs were encoded on the plasmid (Table S2). This plasmid encoded antibiotic resistance, but whether it can be transferred to other bacteria through HGT to lead to the formation of new ARB or even MDRB still needs to be further studied.

### Assessment of ARG horizontal transfer risk in the high-level colistin-resistant strain

To elucidate the multidrug resistance transfer risk and pathogenicity of *R. pickettii* MCR, we analyzed its virulence genes, ARGs and genes related to pathogen and host interactions (Fig. 5d–f). Identification of virulence genes, potential ARGs and genes related to pathogen and host interaction were predicted by examining all coding genes. To ensure that the predicted genes were likely to be homologous, we placed more stringent requirements on the above genes (sequence identity >40%, coverage 90%, *E* value < 1e−10 and score > 200) [24, 25]. A total of 131 potential virulence genes were predicted, which were divided into four types of virulence factors, including offensive virulence factors, defensive virulence factors, factors associated with regulation of virulence-associated genes and nonspecific virulence factors (Fig. 5d). Given that *R. pickettii* MCR carries such a high number of virulence factors, it is assumed that it can pose a toxicological threat to human cells. In addition, a total of 38 potential ARGs of *R. pickettii* MCR were predicted, many of which were multidrug resistance genes (Fig. 5e). The resistance targets for these ARGs covered multiple classes of antibiotics, including rifamycin, fluoroquinolone, peptide, diaminopyrimidine, aminoglycoside, cephalosporin, macrolide, tetracycline, triclosan, aminocoumarin, carbapenem, monobactam, penam, penem, phenicol, sulfonamide, nitroimidazole, glycylcycline, acridine dye and cephamycin (Table S3). The most common resistance mechanism of these ARGs is antibiotic efflux, which may be one of the keys to the multidrug resistance of *R. pickettii* MCR. In addition, we predicted genes related to pathogen and host interactions. A total of 317 potential related genes in *R. pickettii* MCR were predicted and divided into eight types, including genes associated with reduced virulence, unaffected pathogenicity, increased virulence (hypervirulence), loss of pathogenicity, lethal, effector (plant avirulence determinant), chemistry target: resistance to chemical and chemistry target: sensitivity to chemical (Fig. 5f). This indicates that *R. pickettii* MCR is likely pathogenic to the host according to the pathogen and host interaction-related genes it has carried.

**Fig. 6:**
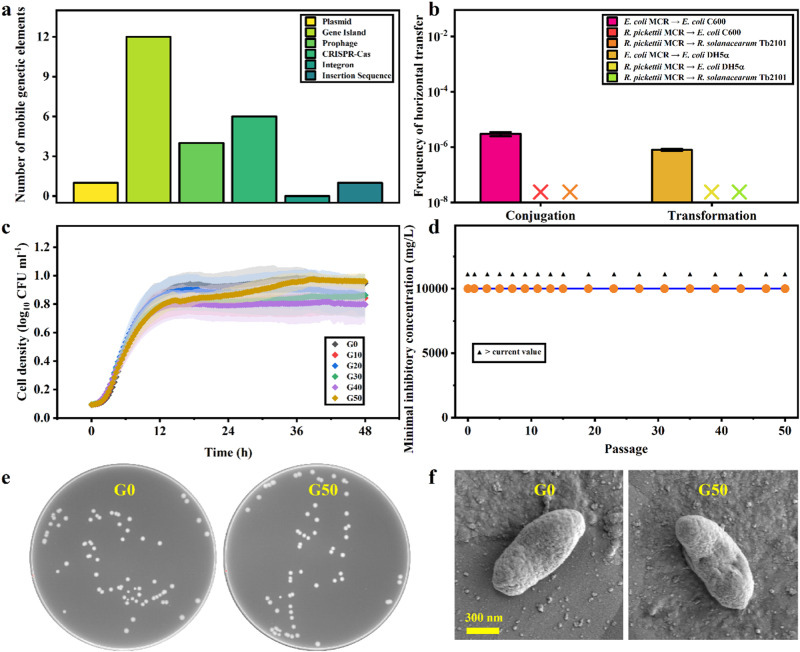
Horizontal and vertical transfer risk analysis of colistin resistance of *Ralstonia pickettii* MCR. **a** Quantitative analysis of MGEs. **b** Conjugative and transformative transfer experiments. **c** Growth curves of wild types and different generations. **d** Colistin resistance in different generations. **e** Colony phenotypes of the first (G0) and 50th generation (G50). **f** Single bacterial phenotypes of G0 and G50.

Furthermore, we explored the transfer risk of high-level colistin resistance of *R. pickettii* MCR by assessing the number of MGEs, intragenus and intergenus conjugative transfer, intragenus and intergenus transformation, and resistance changes in recipients (Fig. 6). The six common MGEs of *R. pickettii* MCR could be identified from the genome information (Fig. 6a). *R. pickettii* MCR carries only one plasmid, in which *traB*, *traD*, *traI*, *trbB*, *trbD*, *trbE*, *trbF*, *trbG*, *trbI*, and *pilV* are found (Table S2), but it does not carry the initiation site *oriT* for plasmid conjugative transfer. From this perspective, this plasmid may be a nonconjugative transfer plasmid. In addition, genome islands, prophages, CRISPR/Cas systems, integrons and insertion sequences are the most important forms of MGEs [26–30], and 12 genome islands were screened from *R. pickettii* MCR. The specific information and distribution of all genome islands are shown in Table S4, and the linear map is shown in Fig. S2a. A total of four prophages were screened from *R. pickettii* MCR (Table S5 and Fig. S2b), and a total of six CRISPR/Cas systems appeared in the *R. pickettii* MCR genome (Table S6 and Fig. S2c). Fortunately, the genome did not have any integron, and there was only one insertion sequence in the *R. pickettii* MCR genome (Table S7 and Fig. S2d). Therefore, there were multiple MGEs in *R. pickettii* MCR, which may be a prerequisite for the transfer of high-level colistin resistance.

Finally, we experimentally verified the nontransferability of the plasmid pRp12D02 (Fig. 6b). The plasmid pRp12D02 could not be transferred by intragenus and intergenus conjugation transfer. In addition, we also tested whether *R. pickettii* MCR could transfer colistin resistance to the recipient by transformation experiments with plasmid DNA. As Fig. 6b shows, pRp12D02 could not transfer colistin resistance to the recipient, via neither intragenus or intergenus transfer. Overall, the plasmid of *R. pickettii* MCR could not transmit its high level of colistin resistance by conjugation or transformation. Subsequently, we experimentally verified the vertical transfer stability of colistin resistance of *R. pickettii* MCR. The growth curves of each of the ten generations reflected the stable vertical proliferation of *R. pickettii* (Fig. 6c). Accordingly, a change in colistin resistance in offspring during passage was not observed, indicating that the high level of colistin resistance of *R. pickettii* could be transmitted stably vertically (Fig. 6d). The colony and single bacterial cell phenotypes of the 50th generation were not different from those of generation 0, which also supported the conclusions above (Fig. 6e, f). Similarly, no significant difference in fitness costs was observed between the generation 0 and 50th generation. Therefore, the risk of horizontal transfer of the high-level colistin resistance of *R. pickettii* MCR was low, but the colistin resistance could be steadily transmitted to offspring through vertical transfer.

## Discussion

### Diverse and abundant ARGs, MGEs, and MDRB facilitate the transmission of antibiotic resistance

Very diverse ARGs were detected with different abundances in all water samples, suggesting the prevalence of ARGs in the over 5000 km aquaculture environment. This might be mainly associated with the intensive use of antibiotics in aquaculture. Previous studies reported high multiantibiotic resistance indices for aquaculture-associated bacteria and various ARGs in aquaculture water [20, 31], suggesting that antibiotics are widely used in the aquaculture industry. Cefotaxime, erythrocin, ofloxacin, tetracycline, lincomycin, and sulfisoxazole were reported to have been commonly used on aquaculture farms in China between 2008 and 2018 [32]. Surprisingly, vancomycin, imipenem, and meropenem are rarely used in aquaculture, but the corresponding ARGs with different abundances were also detected in this study. Vancomycin is often considered the last line of defense against Gram-positive bacteria such as *Streptococcus pneumoniae* and *Enterococcus*, some of which are resistant to most other antibiotics [33]. The widespread distribution of imipenem, and meropenem resistance genes is likely to make it more difficult to treat bacteria harboring extended-spectrum β-lactamases (ESBLs), which hydrolyze almost all antibiotic β-lactams except carbapenems [34]. It has been reported that colistin was used in aquaculture in Vietnam, which may have accelerated the transmission of the colistin resistance gene *mcr* [16]. Similarly, *mcr−1* has been shown to be prevalent in livestock and poultry, and plasmids carrying *mcr−1* have been identified [17]. Cabello et al. suggested that aquaculture is a potential hotspot reservoir for *mcr* [35]. They also pointed out aquaculture as another environmental gateway to the development and globalization of antimicrobial resistance [36]. Although *mcr−1* was also prevalent in aquaculture in this study, there were no reports of the use of colistin in local aquacultural farms. It is speculated that colistin, vancomycin, imipenem and meropenem resistance genes may have been derived from the surrounding environment, such as livestock and poultry breeding, or via the use of organic fertilizers. Indeed, several studies have reported the use of colistin in livestock and poultry production in China and the high prevalence of *mcr−1* in *E. coli* isolated from meat and chicken in slaughterhouses and supermarkets [17, 37]. In addition, due to the proximity of poultry and fish cultivation, aquaculture animals often obtain nutrients from poultry feces [38]. The use of poultry manure as organic fertilizers may also allow ARGs to enter the soil and eventually aquaculture water. Cabello et al. also speculated that the *mcr* determinants (phosphoethanolamine transferase) probably originated primarily or simultaneously in the aquatic environment as a result of aquaculture activities [39]. Therefore, the aquaculture environment is likely to be one of the sinks as well as sources of *mcr−1*. A high abundance of *intI1* was also observed in the aquaculture environment [20, 31, 40], further suggesting that the aquaculture environment is likely to be a source of enrichment and transmission of ARGs. *IntI1* abundance reflects the general response of bacterial communities to selection imposed by anthropogenic pollution, as resistance determinants confer a selective advantage on the bacterial cells that carry them [29]. The high abundance of *intI1*, as a proxy for anthropogenic pollution, supported the possible contamination of aquacultural farms by nearby poultry breeding.

Large amounts of antibiotics used for fish in aquaculture have resulted in the selection of pathogenic MDRB [41]. Due to the types of antibiotics used, the bacterial taxa of culturable MDRB were different among the water samples in this study. Furthermore, this led to differences in the contamination and prevalence characteristics of ARGs in various farming systems. Li et al. revealed that MDRB in the aquaculture environment may be enriched in the human body through the food chain, posing a major threat to aquaculture and human health [42]. Nadella et al. isolated 160 strains of MDRB from *Penaeus vannamei*, and found that 46.2% of the strains carried *intI1*, revealing the wide distribution and potential transfer of MDRB in shrimp farms [43]. The high abundance of MDRB also implied the presence of higher concentrations and diversity of ARGs, which facilitated the transmission of antibiotic resistance. In addition, multidrug resistance genes have greater potential for transfer because they are more frequently carried by MGEs [44]. In conclusion, diverse and abundant of ARGs, MGEs, and MDRB facilitate the transmission of antibiotic resistance in aquaculture.

### HGT contributes to the spread of antibiotic resistance in aquaculture environments

Based on the conjugation transfer model established in this study, we confirmed that antibiotic resistance can be transferred and spread through conjugation in aquaculture water. Similarly, a study demonstrated conjugative transfer efficiencies ranging from 1.1 × 10^−9^ to 1.8 × 10^−3^ for ARGs and MGEs isolated from aquaculture environments [43]. He et al. reported a positivity rate of as high as 92.3% for integral and conjugative elements in 126 strains of *Vibrio cholerae* isolated from aquatic products and environments in Shanghai, suggesting the ubiquity of ARG transfer through conjugation in aquaculture environments [45]. In addition, it was reported that coastal mudflats are reservoirs of extracellular ARGs, which are significantly higher than intracellular ARGs [46]. The high abundance of extracellular ARGs would facilitate the spread of antibiotic resistance via natural transformation. For example, a previous study reported natural transformation of *Vibrio parahaemolyticus* via pVA1 plasmid acquisition as a potential mechanism causing aquatic disease [47]. Our study also showed the high spread efficiency of extracellular ARGs through transformation in aquaculture environments. Collectively, the results of this study offer empirical evidence that antibiotic resistance could spread to recipients and induce the emergence of new ARB or MDRB via HGT in aquaculture environments.

### Origins of the multidrug and high-level colistin resistance of *R. pickettii* MCR

In terms of the broad spectrum and high level of antibiotic resistance, the isolation of *R. pickettii* MCR harboring high levels of colistin resistance in aquaculture waters poses a relatively high health risk. The origin of multidrug resistance and high-level colistin resistance in *R. pickettii* MCR is likely associated with the fact that bacteria encode many genes related to DNA repair, the SOS response, and efflux, energy metabolism, as well as ARGs. Notably, the plasmid pRp12D02 and two chromosomes of *R. pickettii* MCR did not carry the *mcr−1*. However, *mcr−1* is not the only mechanism mediating colistin resistance. Previous studies have shown that the main mechanism of colistin resistance involves the complete loss of LPS resulting from inactivation of the first three genes of the lipid A biosynthetic pathway (*lpxA*, *lpxC*, and *lpxD* genes) [48, 49]. Modifications of target LPS driven by the addition of phosphoethanolamine (PEtN) moieties to lipid A mediated by the chromosomal *pmrCAB* operon can also lead to colistin resistance [48]. In addition, colistin resistance can also be caused by amino acid changes in PmrE, PmrK, and MgrB [50, 51]. Modulation of the two-component regulatory system PhoP/PhoQ also confers colistin resistance [52]. The PhoPQ and PmrAB systems have functions and regulatory pathways that have been found to overlap, so the former is also involved in the mechanism of colistin resistance [53]. In addition, previous studies suggest that efflux genes also contribute to colistin resistance [54, 55]. In this study, the genes encoding PmrA, PmrB, PhoP and multiple efflux pumps as well as *lpxA*, *lpxC*, and *lpxD* also existed on the chromosomes, and the gene encoding PhoP was also located on the plasmid of *R. pickettii* MCR. Therefore, it is assumed that colistin resistance in *R. pickettii* is associated with multiple mechanisms. This finding does not contradict the finding that *mcr−1* is already prevalent in aquaculture environments. For instance, several plasmids carrying *mcr−1* from 17 colistin-resistant strains have been isolated from retail aquatic products, and phages are involved in the incorporation of the *mcr−1* gene into *E. coli* plasmids or chromosomes [56]. Several studies have reported that some transferable plasmids carrying *mcr−1* have been isolated and identified in aquaculture animals [57–59], confirming that this gene can be transmitted. Thus, ample prior evidence confirms the prevalence of *mcr−1* in aquatic environments, although *mcr−1* was not identified from colistin-resistant *R. pickettii* in this study.

### Pathogenicity and transfer risk of antibiotic resistance in the colistin-resistant strain

The strain *R. pickettii* MCR isolated in this study carried multiple virulence genes, ARGs and pathogen‒host interaction-related genes, which undoubtedly raise the health risk of *R. pickettii* MCR (Fig. 5). In addition, *R. pickettii* MCR harbors ARGs related to many types of antibiotics that are currently routinely used in clinical practice. Notably, the *mcr* gene was not detected on the plasmid pRp12D02, but many non-*mcr* genes involved in colistin were found on the plasmid. Although there were multiple conjugation-related genes, such as *traB*, on the plasmid pRp12D02, it lacked the initiation site *oriT* for plasmid transfer. A transferable plasmid should generally have both *tra* and *trb* encoding sexual fimbriae biosynthesis [60], and the initiation site *oriT* for plasmid transfer [61]. From this point of view, the pRp12D02 might be a nontransferable plasmid. Our HGT experiments demonstrated that the plasmid pRp12D02 was unable to transfer colistin resistance to the recipient bacteria through either conjugation or transformation. However, the clonal stability of colistin resistance was shown, and the high level of colistin resistance of *R. pickettii* could be steadily transmitted to offspring through vertical transfer. *R. pickettii* has been isolated from several aquatic products [62, 63]. Although no information is available about the pathogenicity of *R. pickettii* to aquatic animals, numerous studies have confirmed that this strain is a human pathogen [64, 65]. *R. pickettii* has been shown to cause infections in hospital settings, such as bacteremia and meningitis [64]. The strain has been recovered from a wide variety of clinical specimens, including blood, urine, and cerebrospinal fluid [66]. Therefore, carrying genetically stable high levels of colistin and multidrug resistance makes *R. pickettii* a greater threat to human health.

Furthermore, *R. pickettii* MCR carries multiple MGEs, including genome islands, prophages, and insertion sequences, which might also cause the potential spread of high-level colistin resistance. It is generally believed that mild phages can enter the lysogenic cycle, during which the phage genome integrates into the bacterial chromosome to form prophages [67]. In particular, considering that four prophages were integrated into the genome of *R. pickettii* MCR, it is speculated that *R. pickettii* MCR might undergo the transduction process and transfer antibiotic resistance to other bacteria. Further studies are warranted to assess the risk of multidrug resistance or high levels of colistin resistance spreading to other bacteria through phage-mediated transduction.

### Implications of this study

Overall, this study investigated the prevalence features and transmission risk of typical ARGs (especially colistin resistance) and MDRB from over 5000 km in the subtropical coastal aquaculture region. The diverse and abundant ARGs, MGEs, and MDRB met the prerequisites for the spread of antibiotic resistance. In addition, a strain of *R. pickettii* MCR with high-level colistin resistance was isolated. The risk of horizontal transfer of the high-level colistin resistance of *R. pickettii* MCR was low, but colistin resistance could be steadily transmitted to offspring through vertical transfer. However, *mcr−1* was not identified from the genome of *R. pickettii* MCR, suggesting that the prevalence of *mcr−1* in aquaculture environments may be caused by the cobreeding of poultry. The findings underline the importance of One Health approaches to tackling antibiotic resistance. The findings of this study, therefore, indicate the emerging need to respect sustainability, decrease the use of antibiotics in different contexts and promote the use of new antimicrobials and possibly those of natural origin. In addition, land and aquatic food animals should be kept in separate environments to avoid the cross-spread of antibiotic resistance. Likewise, multidisciplinary and intersectorial collaborative efforts are needed to counter the transmission of antibiotic resistance.

There are some limitations in this study that warrant further investigation in the future. For instance, it would be worthwhile to assess seasonal variations in gene abundance, considering that aquaculture water is replaced with fresh seawater regularly. In addition, although the colistin resistance gene *mcr−1* has become prevalent in aquaculture environments, the use of colistin in local aquaculture farms was not documented. Further confirmation of whether the prevalence of *mcr−1* was due to the cobreeding of poultry is recommended.

### Supplementary information

Supplementary information

## Data Availability

Bacterial-genome sequences and 16S rRNA gene sequencing data are available at the US National Center for Biotechnology Information (NCBI; Bethesda, MD, USA; BioProject: PRJNA877814 and PRJNA884867).
