# Supplementary material for: Prevalence and transmission risk of colistin and multidrug resistance in long-distance coastal aquaculture
Source: ISME Commun. 2023 Nov 7;3:115. doi: 10.1038/s43705-023-00321-w (PMC10630474; doi:10.1038/s43705-023-00321-w)
Supplement: Supplementary file 1 — Supplementary information [file 43705_2023_321_MOESM1_ESM.docx]

**Supplementary information (SI)**

**Prevalence and transmission risk of colistin and multidrug resistance in long-distance coastal aquaculture**

**Taicheng An^1,2,^*, Yiwei Cai^1,2^, Guiying Li^1,2^, Shaoting Li^3^, Po Keung Wong^1,2^, Jianhua Guo^4,^*, Huijun Zhao^5^**

^1^ *Guangdong-Hong Kong-Macao Joint Laboratory for Contaminants Exposure and Health, Guangdong Key Laboratory of Environmental Catalysis and Health Risk Control, Institute of Environmental Health and Pollution control, Guangdong University of Technology, Guangzhou 510006, China;*

*^2^* *Guangzhou Key Laboratory of Environmental Catalysis and Pollution Control, Key Laboratory of City Cluster Environmental Safety and Green Development* *of the Ministry of Education, School of Environmental Science and Engineering, Guangdong University of Technology, Guangzhou 510006, China.*

*^3^* *College of Biological and Pharmaceutical Science, Guangdong University of Technology, Guangzhou 510006, China.*

*^4^* *Australian Centre for Water and Environmental Biotechnology, The University of Queensland, St. Lucia, Brisbane, QLD 4072, Australia.*

*^5^* *Centre for Clean Environment and Energy, and Griffith School of Environment, Gold Coast Campus, Griffith University, Queensland 4222, Australia.*

**Corresponding Author:** Prof. Taicheng An, E-mail: [antc99@gdut.edu.cn](mailto:antc99@gdut.edu.cn); Prof. Jianhua Guo, E-mail: jianhua.guo@uq.edu.au

**Text S1**

**DNA extraction and qPCR analysis**

DNA from the membranes was extracted using the Soil DNA Rapid Extraction Kit (Sangon Biotech, China) and stored at -20°C [1]. The expression of ARGs (*blaCTX-M*, *blaIMP*, *blaKPC*, *blaNDM*, *aphA*, *mcr-1*, *vanA*, *ermB*, *tetW*, *cmlA*, *mefA*, *rpoB*, *sul1*, and *qnrB*) and *intI1* was quantified using qPCR. The danger levels of antibiotics corresponding to the ARGs are shown in Table S1. Bacterial 16S rRNA was quantified using the 27 F and 1492R pair that was used as an internal control for data normalization. The qPCR was performed with SYBR Green qPCR Mix (Monad, China) in a thermal cycler (CFX 96 Touch system, Bio-Rad, USA). Detailed ARGs and 16S rRNA design primers and reference information are given in Table S8.

The qPCR mix (20 μL) consists of 10 μL of SYBR Green qPCR Mix, 1 μL of forward and reverse primers (final concentration 0.5 μm), 2 μL of DNA template, and 6 μL of nucleic acid-free water. A previous study was referenced by the programming of the thermal cycler [2]. The expression levels of related genes were calculated using the 2^-ΔΔCT^ method [3].

**Text S2**

**Plasmid extraction**

The plasmids of the proliferated bacterial colonies were extracted using the UNIQ-500 column plasmid DNA mass extraction kit (Sangon Biotech, China). According to the experimental operation in the manual, 50 mL of bacterial solution was taken, centrifuged at 10,000 rpm for 3 min, the bacterial cells were collected, and 10 mL of Buffer P1 was added to the bacterial cell pellet to completely suspend the bacterial cells. Ten mL of Buffer P2 was added, mixed well, and allowed to stand at room temperature for 2-4 min. Fourteen mL of Buffer P3 was added, immediately inverted 5-10 times, and left at room temperature for 5 min, 90 °C water bath for 8 min, then placed at -20 °C for 10 min, centrifuged at 12,000 rpm for 15 min. All the supernatant was transferred to the adsorption column, placed at room temperature for 5 min, and centrifuged at 8,000 rpm for 2 min. 5 mL of Buffer DW1 was added to the adsorption column and centrifuged at 8,000 rpm for 2 min. The liquid in the collection tube was discarded and the adsorption column was placed in the same collection tube. 5 mL of Wash Solution was added to the adsorption column for two washes and centrifuged at 8,000 rpm for 2 min. The empty adsorption column and collection tube were placed in a centrifuge and centrifuged at 10,000 rpm for 2 min. The adsorption column was put into a clean 50 mL centrifuge tube, 500 μL of Elution Buffer was added to the center of the adsorption membrane, left standing at room temperature for 2 min, and centrifuged at 10,000 rpm for 2 min. The resulting plasmid DNA solution was stored at -20 °C or used for subsequent experiments.

**Text S3**

**MDRB diversity analysis**

One % agarose gel was used to detect the extracted genomic DNA by electrophoresis. According to the designated sequencing region, specific primers with barcode were synthesized. PCR experiments were performed on an ABI GeneAmp^®^ 9700 PCR Amplifier (Applied Biosystems, USA) using TransGen AP221-02 (TransGen Biotech, China) and TransStart Fastpfu DNA Polymerase Kit (TransGen Biotech, China). The PCR products of the same sample were mixed and detected by 2% agarose gel electrophoresis, and the PCR products were recovered by cutting the gel using the AxyPrepDNA gel recovery kit (Axygen, USA), and eluted with Tris-HCl; 2% agarose electrophoresis was used for detection. Referring to the preliminary quantitative results of electrophoresis, the PCR products were detected and quantified by QuantiFluor™-ST blue fluorescence quantitative system (Promega, USA), and then mixed in corresponding proportions according to the requirements of the sequencing amount of each sample. Miseq library construction: 1) The official Illumina adapter sequence was added to the outer end of the target region by PCR; 2) The PCR product was recovered by cutting the gel using a gel recovery kit; 3) Tris-HCl buffer was used for elution, and 2% agarose was used for electrophoresis detection; 4) Single-stranded DNA fragments were generated by using TruSeq^TM^ DNA Sample Prep Kit (Illumine, USA) and sodium hydroxide for denaturation. Miseq sequencing: 1) One end of the DNA fragment was complementary to the primer base and fixed on the chip; 2) Using the DNA fragment as a template, the base sequences fixed on the chip are used as primers for PCR synthesis, and the target DNA fragment to be tested was synthesized on the chip; 3) After denaturation and annealing, the other end of the DNA fragment on the chip was randomly complementary to another nearby primer, and was also fixed to form a "bridge"; 4) DNA clusters were generated by PCR amplification; 5) DNA amplicons were linearized into single strands; 6) By adding the modified DNA polymerase and dNTPs with 4 kinds of fluorescent labels, only one base was synthesized in each cycle; 7) The surface of the reaction plate was scanned with a laser to read the nucleotide species polymerized in the first round of the reaction of each template sequence; 8) The "fluorophore" and "termination group" were chemically cleaved to restore the 3' end stickiness, and the second nucleotide continues to be polymerized; 9) The fluorescence signal results collected in each round were counted to obtain the sequence of the template DNA fragment. Relevant technical services were provided by Shanghai Majorbio Bio-pharm Technology (Shanghai, China).

**Text S4**

**Identification and genome analysis of the** **high-level colistin-resistant strain**

The obtained raw sequences at both ends were subjected to quality control to remove low-quality bases to obtain clean sequences. The spliced assembly sequence was blasted with the NT database to obtain the species information of the top 10 species with the highest similarity, and the aligned species with the highest similarity was selected as the result of the bacterial identification of this project.

The high-level colistin-resistant strain was identified as *Ralstonia pickettii*, named *R. pickettii* MCR, and its genome was analyzed. Through the sequencing method of Illumina Hiseq+PacBio (related technical services were provided by Shanghai Majorbio Bio-pharm Technology, Shanghai, China), PacBio sequencing data and 100× Illumina sequencing data of no less than 100× of the genome were provided, the loss of small plasmid (<15kb) information was avoided, and the complete genome containing the plasmid was obtained.

**Text S5**

**Whole-genome sequencing of the high-level colistin-resistant strain**

*Illumina Hiseq sequencing experiment flow*: 1. Library construction: 1) Purified genomic DNA was collected; 2) The genomic DNA was fragmented by Covaris to construct a genomic sequencing library; 3) A & B adapters were ligated; 4) The self-ligated fragments of the adapters were screened and removed; 5) Agarose gel electrophoresis was used to screen fragments, and the fragments with A linker at one end and B linker at the other end were retained; 6) Denaturation with sodium hydroxide, resulting in single-stranded DNA fragments. 2. Bridge PCR: 1) One end of the DNA fragment was complementary to the primer base and fixed on the chip; 2) The other end is randomly complementary to another nearby primer, and is also fixed to form a "bridge"; 3) DNA clusters were generated by PCR amplification; 4) The DNA amplicons were linearized into single strands; 5) By adding the modified DNA polymerase and dNTPs with 4 kinds of fluorescent labels, only one base was synthesized in each cycle; 6) The surface of the reaction plate was scanned with a laser to read the nucleotide species polymerized in the first round of the reaction of each template sequence; 8) The "fluorophore" and "termination group" were chemically cleaved to restore the 3' end stickiness, and the second nucleotide continues to be polymerized; 9) The fluorescence signal results collected in each round were counted to obtain the sequence of the template DNA fragment.

*Single-molecule PacBio sequencing experimental process*: 1. DNA purification and detection: Nanodrop2500 was used to detect the concentration of genomic DNA to ensure that the quality of DNA for subsequent experiments was high enough (no degradation, OD260/280=1.8-2.0, and the total amount was not less than 10 μg). 2. Single-molecule sequencing library construction: 1) Fragmentation: The genomic DNA was processed into 8-10 k fragments by G-tubes; 2) The ends were blunted, and the two ends were connected to the circular single chain: The two ends of the single chain were respectively connected to the double-stranded positive and negative chains to obtain a structure similar to a dumbbell ("horse loop"), which was called SMRT Bell. 3. Single-molecule sequencing: 1) The single-stranded loop of the library was annealed and bound to the polymerase at the bottom of the immobilized ZMW (zero-mode waveguides). 2) Sequenced after binding was complete. 4. Single-molecule sequencing quality assessment: Similar to the single-base quality representation method in second-generation sequencing, 90% accuracy was represented by Q10, 99% accuracy was represented by Q20, and so on. Relevant technical services were provided by Shanghai Majorbio Bio-pharm Technology (Shanghai, China).

**Text S6**

**Antibiotic resistance evolution and recovery experiments**

After multi-drug resistance profiling of *R. pickettii* MCR, it was found that its resistance to colistin is at an ultra-high level. However, ARB face a variety of stressors in the environment, which may lead to further enhancement or evolution of antibiotic resistance. Therefore, it is of great significance to explore the potential of bacteria to enhance antibiotic resistance under specific stress conditions. In this study, photocatalysis was used as the stressor and a 50 mL photocatalytic reactor was used as the induction reactor. TiO_2_ nanotubes were used as photocatalysts, and their preparation methods and main parameters were reported in an earlier article [4]。The light source used in this experiment is a 365 nm LED lamp with a light intensity of 24 mW cm^−2^. The bacteria cultured overnight was centrifuged at 8000 rpm for 2 min in a high-speed refrigerated centrifuge to remove the medium components, and washed twice with normal saline The bacteria obtained by centrifugation were diluted with physiological saline to a concentration of about 1-1.5 × 10^8^ CFU mL^-1^ in the suspension system. Photocatalytically induced bacterial suspension samples at different times were serially diluted with physiological saline to the appropriate bacterial concentration. 100 μL of the final diluted bacterial solution was evenly spread on the sterilized nutrient agar medium, placed in an electric thermostatic incubator at 37 °C for 24 h, and the number of persistent small colonies and total colonies were recorded.

The passage recovery experiment was carried out on the persistent small colony variants (SCVs) of *R. pickettii* MCR after induction for 8 h, and the colistin resistance of each generation of progeny strains was determined by the micro-broth dilution method. For specific operations, refer to the above experimental method for multidrug resistance profiling analysis. In addition to monitoring the recovery of resistance in each progeny during the passage, the recovery of the phenotype of each progeny was also observed. Each progeny colony was photographed on a gel imaging system (Gel Doc™ XR+, Bio-Rad, USA) using the SYBR Green Application program, and images were obtained using Image J (Version 1.52，https://imagej.net/Welcome) to measure the diameter of the colonies. The same procedure was used for the characterization of single bacteria.

**Text S7**

**Conjugation and transformation experiments**

Streptomycin-resistant *E. coli* C600 from a different genus and *Ralstonia solanacearum* (*R. s* Tb2101, from South China University of Technology) from the same genus were used as recipient bacteria, and *R. pickettii* MCR after overnight culture was used as donor bacteria to explore the potential of intra- and inter-genus conjugative transfer. The colistin-resistant *E. coli* MCR was used as the control group. The general experimental operation process refers to the conjugation transfer experiment in the manuscript. The main difference is: When studying intra- genus conjugative transfer, 100 μL of the diluted bacterial solution should be spread on TTC Tergitol agar supplemented with 2048 mg L^-1^ colistin, while the recipient bacteria in the control group should be spread on the TTC Tergitol agar containing no antibiotics.

Plasmids of *R. pickettii* MCR were extracted using the NucleoBond BAC 100 Large Plasmid Extraction Kit (MACHEREY-NAGEL GmbH & Co. KG, Germany). The extraction procedure was briefly described as follows: bacteria in logarithmic growth phase were centrifuged at 6000 x g for 15 min at 4 °C, and 24 mL of RNase-supplemented Buffer S1 was added to resuspend the bacteria. Then 24 mL of Buffer S2 was added, mixed by inversion 6-8 times, and incubated at room temperature (ca. 25 °C) for 3 min. 24 mL of 4 ℃ pre-cooled Buffer S3 was added, mixed by inversion 6-8 times, and incubated on ice for 5 min. 6 mL of Buffer N2 was used to equilibrate the column and the waste was discarded. The NucleoBond pleated filter was placed in the funnel, the filter was moistened with a few drops of Buffer N2, and the bacterial lysate was poured into the filter. The collected liquid was transferred to the NucleoBond column, and the waste liquid was discarded after all the liquid passed through the column. 18 mL of Buffer N3 was used to wash the column twice, followed by elution of plasmid DNA with 15 mL of Buffer N5 pre-warmed at 50 °C. Then 11 mL of isopropanol was added, and after standing for 5 min, centrifuged at 10,000 x g for 30 min, and the supernatant was carefully discarded. 5 mL of 70% ethanol was added and centrifuged at 10,000 x g for 10 min. After blotting the ethanol, the plasmid DNA was naturally dried at room temperature. 300 μL of TE Buffer was added to dissolve the plasmid DNA.

Competent *E. coli* DH5α and *R. s* Tb2101 were used as recipient bacteria for inter- and intra-genus transformation, wherein competent *R. s* Tb2101 was prepared with a Super-competent Cell Preparation Kit (Sangon Biotech, China).The preparation process was slightly modified according to the instructions: 500 μl of the overnight cultured *R. s* Tb2101 bacterial solution was inoculated into 50 mL of BT Media, and the culture was shaken at 30 °C on a shaker until the bacterial solution OD600 = 0.5-0.6. The bacterial solution was transferred to a 50 mL polypropylene plastic centrifuge tube and placed on ice for 5-10 min. The bacterial pellet was collected by centrifugation at 3,500-5000 rpm for 5 min at 4°C. The bacteria were gently and thoroughly resuspended in 16 mL of ice-cold BT Buffer A, and placed on ice for 10-15 min. The bacterial pellet was collected by centrifugation at 3,500-5,000 rpm for 2-5 min at 4°C. The bacteria were gently and fully resuspended in 4 mL of ice-cold BT Buffer B, and aliquoted 100 μl per tube into 1.5-mL centrifuge tubes pre-cooled on ice. Bacteria were directly used for transformation or frozen in liquid nitrogen and stored in an ultra-low temperature refrigerator. Transformation protocols refer to the plasmid transformation experiments of the manuscript.

**Table S1. Classes, abbreviations and danger levels of antibiotics corresponding to the ARGs of interest in this study.**

| **Classes** | **Sections** | **Antibiotics** | **Abbreviation** | **MIC of *E. coli*** | **Danger Level** |
| --- | --- | --- | --- | --- | --- |
| β-lactam | Cephalosporins | cefotaxime | CTX | 2 | Emergency |
|  | Carbapenems | imipenem | IMP | 8 | Emergency |
|  |  | meropenem | MEM | 8 | Emergency |
| Aminoglycosides |  | kanamycin | KAN | 2 | Threaten |
| Polypeptide |  | colistin | PE | 2 | **Last defense** |
|  |  | vancomycin | VAN | - | Threaten |
| Lincosamides |  | lincomycin | LIN | - | Ordinary |
| Tetracyclines |  | tetracycline | TET | - | Ordinary |
| Chloramphenicols |  | chloramphenicol | CHL | 8 | Ordinary |
| Macrolides |  | erythrocin | ERY | - | Attention |
| Rifamycins |  | rifampicin | RIF | - | Ordinary |
| Sulfonamides |  | sulfisoxazole | SIX | - | Ordinary |
| Quinolones |  | ofloxacin | OFX | 0.5 | Threaten |

**Table S2. Conjugation-related genes and ARGs on plasmids**

| **Function** | **Gene** | **Gene ID** | **Description** |
| --- | --- | --- | --- |
| Conjugation-related genes | *traB* | pA_gene0037 | TraB/GumN family protein |
|  | *traD* | pA_gene0016 | conjugative transfer system coupling protein TraD |
|  | *traI* | pA_gene0015 | TraI domain-containing protein |
|  | *trbB* | pA_gene0037 | cnjugal transfer protein TrbB |
|  | *trbD* | pA_gene0011 | VirB3 family type IV secretion system protein |
|  | *trbE* | pA_gene0012 | type IV secretory pathway VirB4 components-like protein |
|  | *trbF* | pA_gene0008 | type IV secretion system protein |
|  | *trbG* | pA_gene0007 | pobable conjugal transfer protein TrbG |
|  | *trbI* | pA_gene0006 | cnjugal transfer protein TrbI |
|  | *pilV* | pA_gene0337 | shufflon system plasmid conjugative transfer pilus tip adhesin PilV |
| ARGs | *vanSF* | pA_gene0059 | glycopeptide antibiotic |
|  | *vanSM* | pA_gene0140/ pA_gene0221 | glycopeptide antibiotic |
|  | *arlR* | pA_gene0060/ pA_gene0220 | acridine dye;fluoroquinolone antibiotic |
|  | *amrB* | pA_gene0115 | aminoglycoside antibiotic |
|  | *mtrA* | pA_gene0141/ pA_gene0167 | macrolide antibiotic;penam |
|  | *adeB* | pA_gene0143 | glycylcycline;tetracycline antibiotic |
|  | *macA* | pA_gene0144 | macrolide antibiotic |
|  | *macB* | pA_gene0246 | macrolide antibiotic |
|  | *novA* | pA_gene0156 | aminocoumarin antibiotic |
|  | *msbA* | pA_gene0169 | nitroimidazole antibiotic |
|  | *golS* | pA_gene0198 | carbapenem;cephalosporin; cephamycin;monobactam;penam; penem;phenicol antibiotic |
|  | *tet(30)* | pA_gene0222 | tetracycline antibiotic |
|  | *muxA* | pA_gene0260 | aminocoumarin antibiotic;macrolide antibiotic; monobactam;tetracycline antibiotic |
|  | *mexN* | pA_gene0261 | phenicol antibiotic |

**Table S3. Final identified ARGs in the genome of *R. pickettii* MCR.**

| **Gene ID** | **Location** | **ARO Name** | **ARO Accession** | **Drug Class** | **Resistance Mechanism** |
| --- | --- | --- | --- | --- | --- |
| gene0089 | Chromosome1 | *iri* | 3002884 | rifamycin | antibiotic inactivation |
| gene0512 | Chromosome1 | *AbaQ* | 3004574 | fluoroquinolone | antibiotic efflux |
| gene0628 | Chromosome1 | *bacA* | 3002986 | peptide | antibiotic target alteration |
| gene0819 | Chromosome1 | *dfrA3* | 3003105 | diaminopyrimidine | antibiotic target replacement |
| gene0843 | Chromosome1 | *amrA* | 3002982 | aminoglycoside | antibiotic efflux |
| gene0844 | Chromosome1 | *AxyY* | 3004144 | aminoglycoside; cephalosporin; fluoroquinolone; macrolide | antibiotic efflux |
| gene1313 | Chromosome1 | *emrB* | 3000074 | fluoroquinolone | antibiotic efflux |
| gene1344 | Chromosome1 | *arnA* | 3002985 | peptide | antibiotic target alteration |
| gene1346 | Chromosome1 | *PmrF* | 3003578 | peptide | antibiotic target alteration |
| gene1617 | Chromosome1 | *macB* | 3000535 | macrolide | antibiotic efflux |
| gene1639 | Chromosome1 | *MexK* | 3003693 | macrolide; tetracycline; triclosan | antibiotic efflux |
| gene1645 | Chromosome1 | *CpxR* | 3004054 | aminocoumarin; aminoglycoside; carbapenem; cephalosporin; cephamycin; diaminopyrimidine; fluoroquinolone; macrolide; monobactam; penam; penem; peptide; phenicol; sulfonamide; tetracycline | antibiotic efflux |
| gene1779 | Chromosome1 | *smeR* | 3003066 | aminoglycoside; cephalosporin; cephamycin; penam | antibiotic efflux |
| gene2172 | Chromosome1 | *msbA* | 3003950 | nitroimidazole | antibiotic efflux |
| gene2295 | Chromosome1 | *AbaQ* | 3004574 | fluoroquinolone | antibiotic efflux |
| gene3007 | Chromosome1 | *kdpE* | 3003841 | aminoglycoside | antibiotic efflux |
| gene3061 | Chromosome1 | *adeL* | 3000620 | fluoroquinolone; tetracycline | antibiotic efflux |
| gene3427 | Chromosome1 | *acrB* | 3000216 | cephalosporin; fluoroquinolone; glycylcycline; penam; phenicol; rifamycin; tetracycline; triclosan | antibiotic efflux |
| gene3428 | Chromosome1 | *OprM* | 3000379 | acridine dye; aminocoumarin; aminoglycoside; carbapenem; cephalosporin; cephamycin; diaminopyrimidine; fluoroquinolone; macrolide; monobactam; penam; penem; peptide; phenicol; sulfonamide; tetracycline | antibiotic efflux |
| gene3504 | Chromosome2 | *OXA-22* | 3001417 | cephalosporin; penam | antibiotic inactivation |
| gene3598 | Chromosome2 | *CpxR* | 3004054 | aminocoumarin; aminoglycoside; carbapenem; cephalosporin; cephamycin; diaminopyrimidine; fluoroquinolone; macrolide; monobactam; penam; penem; peptide; phenicol; sulfonamide; tetracycline | antibiotic efflux |
| gene3673 | Chromosome2 | *OXA-444* | 3003600 | cephalosporin; penam | antibiotic inactivation |
| gene3722 | Chromosome2 | *AbaQ* | 3004574 | fluoroquinolone | antibiotic efflux |
| gene3770 | Chromosome2 | *rosB* | 3003049 | peptide | antibiotic efflux |
| gene3944 | Chromosome2 | *OpmB* | 3004072 | aminocoumarin; macrolide; monobactam; tetracycline | antibiotic efflux |
| gene3945 | Chromosome2 | *mdtC* | 3000794 | aminocoumarin | antibiotic efflux |
| gene3946 | Chromosome2 | *MuxB* | 3004074 | aminocoumarin; macrolide; monobactam; tetracycline | antibiotic efflux |
| gene3947 | Chromosome2 | *MuxA* | 3004073 | aminocoumarin; macrolide; monobactam; tetracycline | antibiotic efflux |
| gene3972 | Chromosome2 | *baeS* | 3000829 | aminocoumarin; aminoglycoside | antibiotic efflux |
| gene3973 | Chromosome2 | *baeR* | 3000828 | aminocoumarin; aminoglycoside | antibiotic efflux |
| gene3993 | Chromosome2 | *adeH* | 3000779 | fluoroquinolone; tetracycline | antibiotic efflux |
| gene3994 | Chromosome2 | *ceoB* | 3003010 | aminoglycoside; fluoroquinolone | antibiotic efflux |
| gene3995 | Chromosome2 | *adeG* | 3000778 | fluoroquinolone; tetracycline | antibiotic efflux |
| gene3998 | Chromosome2 | *adeL* | 3000620 | fluoroquinolone; tetracycline | antibiotic efflux |
| gene4531 | Chromosome2 | *rosA* | 3003048 | peptide | antibiotic efflux |
| gene4607 | Chromosome2 | *MexK* | 3003693 | macrolide; tetracycline; triclosan | antibiotic efflux |
| pA_gene0141 | PlasmidA | *mtrA* | 3000816 | macrolide; penam | antibiotic efflux |
| pA_gene0220 | PlasmidA | *arlR* | 3000838 | acridine dye; fluoroquinolone | antibiotic efflux |

**Table S4. All gene islands specific information and distribution.**

| **Location** | **Island ID** | **Sample Name** | **Island Start** | **Island End** | **Length (bp)** | **Method** | **CDS No.** |
| --- | --- | --- | --- | --- | --- | --- | --- |
| Chromosome1 | GI01 | *Ralstonia* | 1180738 | 1188167 | 7429 | IslandPath-DIMOB | 7 |
| Chromosome1 | GI02 | *Ralstonia* | 1206677 | 1220080 | 13403 | IslandPath-DIMOB | 9 |
| Chromosome1 | GI03 | *Ralstonia* | 1876756 | 1914488 | 37732 | IslandPath-DIMOB | 45 |
| Chromosome1 | GI04 | *Ralstonia* | 2120941 | 2142351 | 21410 | IslandPath-DIMOB | 40 |
| Chromosome1 | GI05 | *Ralstonia* | 3063 | 60003 | 56940 | IslandPath-DIMOB | 36 |
| Chromosome1 | GI06 | *Ralstonia* | 3140215 | 3159228 | 19013 | IslandPath-DIMOB | 25 |
| Chromosome1 | GI07 | *Ralstonia* | 3357632 | 3378738 | 21106 | IslandPath-DIMOB | 17 |
| Chromosome1 | GI08 | *Ralstonia* | 986690 | 1099632 | 112942 | IslandPath-DIMOB | 152 |
| Chromosome2 | GI09 | *Ralstonia* | 1032772 | 1061770 | 28998 | IslandPath-DIMOB | 22 |
| PlasmidA | GI10 | *Ralstonia* | 118398 | 122390 | 3992 | IslandPath-DIMOB | 8 |
| PlasmidA | GI11 | *Ralstonia* | 139838 | 145137 | 5299 | IslandPath-DIMOB | 7 |
| PlasmidA | GI12 | *Ralstonia* | 188256 | 216553 | 28297 | IslandPath-DIMOB | 21 |

**Table S5. All prophages specific information and distribution.**

| **Prophage ID** | **Sample Name** | **Location** | **Start** | **End** | **Length (bp)** | **CDS No.** | **Taxonomy** | **GC (%)** |
| --- | --- | --- | --- | --- | --- | --- | --- | --- |
| Ph01 | *Ralstonia* | Chromosome1 | 995908 | 1008030 | 12123 | 14 | *Myoviridae* | 63.75 |
| Ph02 | *Ralstonia* | Chromosome1 | 1064345 | 1092336 | 27992 | 29 | *Myoviridae / Siphoviridae* | 63.75 |
| Ph03 | *Ralstonia* | Chromosome1 | 1876756 | 1912747 | 35992 | 39 | *Myoviridae* | 63.75 |
| Ph04 | *Ralstonia* | Chromosome1 | 2102022 | 2130658 | 28637 | 38 | *Myoviridae* | 63.75 |

**Table S6. All CRISPR/Cas systems specific information and distribution.**

| **Location** | **CRISPR ID** | **Sample Name** | **Start** | **End** | **DR No.** | **DR Average Len (bp)** | **SPA Average Len (bp)** |
| --- | --- | --- | --- | --- | --- | --- | --- |
| PlasmidA | CRISPR6 | *Ralstonia* | 63216 | 63429 | 3 | 46 | 38 |
| Chromosome2 | CRISPR5 | *Ralstonia* | 1207258 | 1207370 | 2 | 32 | 49 |
| Chromosome1 | CRISPR1 | *Ralstonia* | 453143 | 453309 | 3 | 23 | 49 |
| Chromosome1 | CRISPR2 | *Ralstonia* | 1392890 | 1393262 | 6 | 37 | 30 |
| Chromosome1 | CRISPR3 | *Ralstonia* | 1646173 | 1646545 | 6 | 37 | 30 |
| Chromosome1 | CRISPR4 | *Ralstonia* | 2717220 | 2717631 | 5 | 31 | 64 |

**Table S7. All insertion sequence specific information and distribution.**

| **Is ID** | **Location** | **IS Family** | **Group** | **Type** | **Start** | **End** | **Length (bp)** | **Score** | **Evalue** | **CDS No.** |
| --- | --- | --- | --- | --- | --- | --- | --- | --- | --- | --- |
| IS001 | Chromosome1 | IS5 | IS1031 | partial | 1583430 | 1584662 | 1232 | 28 | 3.9E-35 | 1 |

**Table S8. Detailed primer design information for ARGs and 16S rRNA.**

| **Antibiotics** | **ARGs** | **Forward** | **TM (°C)** | **Reverse** | **TM (°C)** | **References** |
| --- | --- | --- | --- | --- | --- | --- |
| **cefotaxime** | ***bla_CTX-M_*** | GGTTGAGGCTGGGTGAAGTA | 57.45 | GACGTTAAACACCGCCATTC | 55.4 | [2] |
| **imipenem** | ***bla_IMP_*** | AAGTTAGTCAMTTGGTTTGTGGAGC | 56.3 | CAAACCACTACGTTATCTKGAGTGTG | 58.02 | [5] |
| **meropenem** | ***bla_KPC_*** | CAGCTCATTCAAGGGCTTTC | 55.4 | GGCGGCGTTATCACTGTATT | 55.4 | [6] |
|  | ***bla_NDM_*** | TTGGCGATCTGGTTTTCC | 52.62 | GGTTGATCTCCTGCTTGA | 52.62 | [5] |
| **kanamycin** | ***aphA*** | CGACGGTAGAGCAAAGGT | 54.9 | AGCGGACAGCATCAGTAA | 52.62 | [7] |
| **colistin** | ***mcr-1*** | CGGTCAGTCCGTTTGTTC | 54.9 | CTTGGTCGGTCTGTAGGG | 57.18 | [2] |
| **vancomycin** | ***vanA*** | CTGTGAGGTCGGTTGTGCG | 59.48 | TTTGGTCCACCTCGCCA | 54.61 | [8] |
| **lincomycin** | ***ermB*** | GATACCGTTTACGAAATTGG | 51.3 | GAATCGAGACTTGAGTGTGC | 55.4 | [9] |
| **tetracycline** | ***tetW*** | GAGAGCCTGCTATATGCCAGC | 59.52 | GGGCGTATCCACAATGTTAAC | 55.61 | [10] |
| **chloramphenicol** | ***cmlA*** | GCCAGCAGTGCCGTTTAT | 54.9 | GGCCACCTCCCAGTAGAA | 57.18 | [11] |
| **erythrocin** | ***mefA*** | AGTATCATTAATCACTAGTGC | 49.76 | TTCTTCTGGTACTAAAAGTGG | 51.71 | [9] |
| **rifampicin** | ***rpoB*** | CGCTGAAGGTGGCGTAAAAC | 57.45 | CTCACCTGGACGCATGACTT | 57.45 | [12] |
| **sulfisoxazole** | ***sul1*** | CGCACCGGAAACATCGCTGCAC | 63.26 | TGAAGTTCCGCCGCAAGGCTCG | 63.26 | [13] |
| **ofloxacin** | ***qnrB*** | GCGACGTTCAGTGGTTCAG | 57.32 | TGTCCAACTTAACGCCTTGTAA | 53.95 | [14] |
|  | ***intI1*** | GGCTTCGTGATGCCTGCTT | 57.32 | CATTCCTGGCCGTGGTTCT | 57.32 | [6] |
|  | ***16S*** | TGCATCTGATACTGGCAAGC | 55.4 | ACCTGAGCGTCAGTCTTCGT | 57.45 | [2] |

**Table S9. The top 10 species information of species similarity in NT database blast.**

| **Description** | **Genus** | **Max Score** | **Total Score** | **Query Cover** | **Per. Ident** | **Accession** |
| --- | --- | --- | --- | --- | --- | --- |
| *Ralstonia* sp. strain M1 16S ribosomal RNA gene, partial sequence | ***Ralstonia*** | 2372 | 2372 | 100 % | 100.00 % | MH844635.1 |
| *Ralstonia* *pickettii* strain 1F 16S ribosomal RNA gene, partial sequence | ***Ralstonia*** | 2372 | 2372 | 100 % | 100.00 % | MK282223.1 |
| *Ralstonia* *pickettii* strain ADZH5101 16S ribosomal RNA gene, partial sequence | ***Ralstonia*** | 2372 | 2372 | 100 % | 100.00 % | MK610811.1 |
| *Ralstonia* *pickettii* strain B16-211 16S ribosomal RNA gene, partial sequence | ***Ralstonia*** | 2372 | 2372 | 100 % | 100.00 % | MK072679.1 |
| *Ralstonia* sp. strain LC 16S ribosomal RNA gene, partial sequence | ***Ralstonia*** | 2372 | 2372 | 100 % | 100.00 % | MK418966.1 |
| *Ralstonia* sp. gene for 16S rRNA, partial sequence, clone: MmiIRO02_GFR01 | ***Ralstonia*** | 2372 | 2372 | 100 % | 100.00 % | LC370594.1 |
| *Ralstonia* sp. gene for 16S rRNA, partial sequence, clone: MmiIRO01_GFR01 | ***Ralstonia*** | 2372 | 2372 | 100 % | 100.00 % | LC370591.1 |
| *Ralstonia* sp. gene for 16S rRNA, partial sequence, clone: HmaTKB01_OGR01 | ***Ralstonia*** | 2372 | 2372 | 100 % | 100.00 % | LC370588.1 |
| *Ralstonia* sp. gene for 16S rRNA, partial sequence, clone: CfaKSD01_GR01 | ***Ralstonia*** | 2372 | 2372 | 100 % | 100.00 % | LC370551.1 |
| *Ralstonia pickettii* strain dek13b 16S ribosomal RNA gene, partial sequence | ***Ralstonia*** | 2372 | 2372 | 100 % | 100.00 % | MF179868.1 |
| *Ralstonia pickettii* strain T3-PYR 16S ribosomal RNA gene, partial sequence | ***Ralstonia*** | 2372 | 2372 | 100 % | 100.00 % | KY241477.1 |


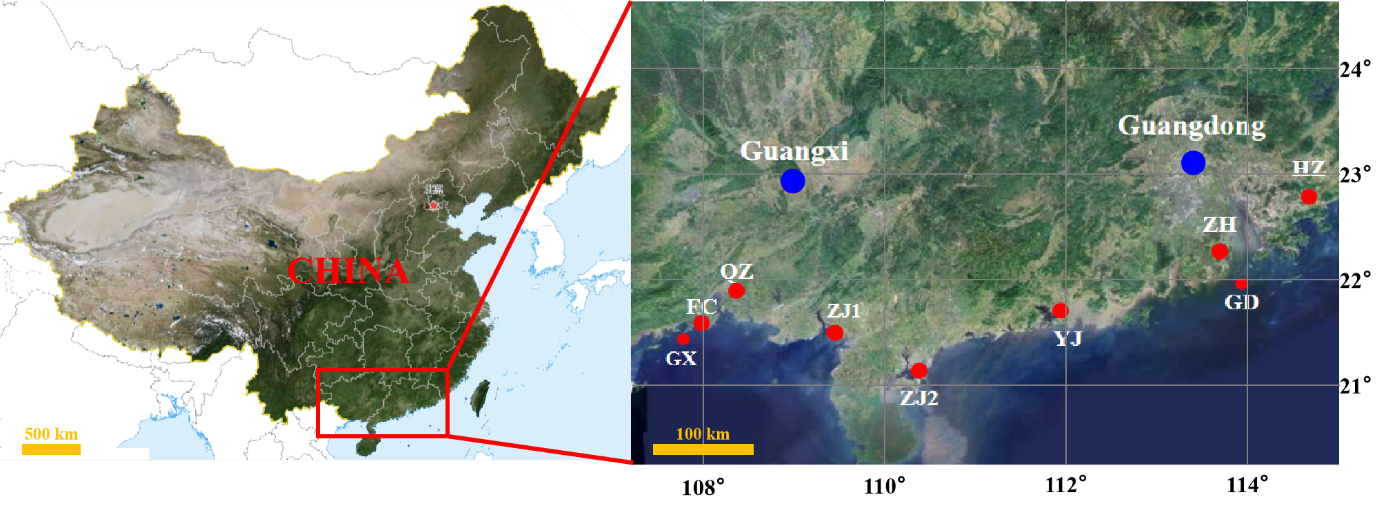


**Fig. S1.** Information and distribution of sampling locations.


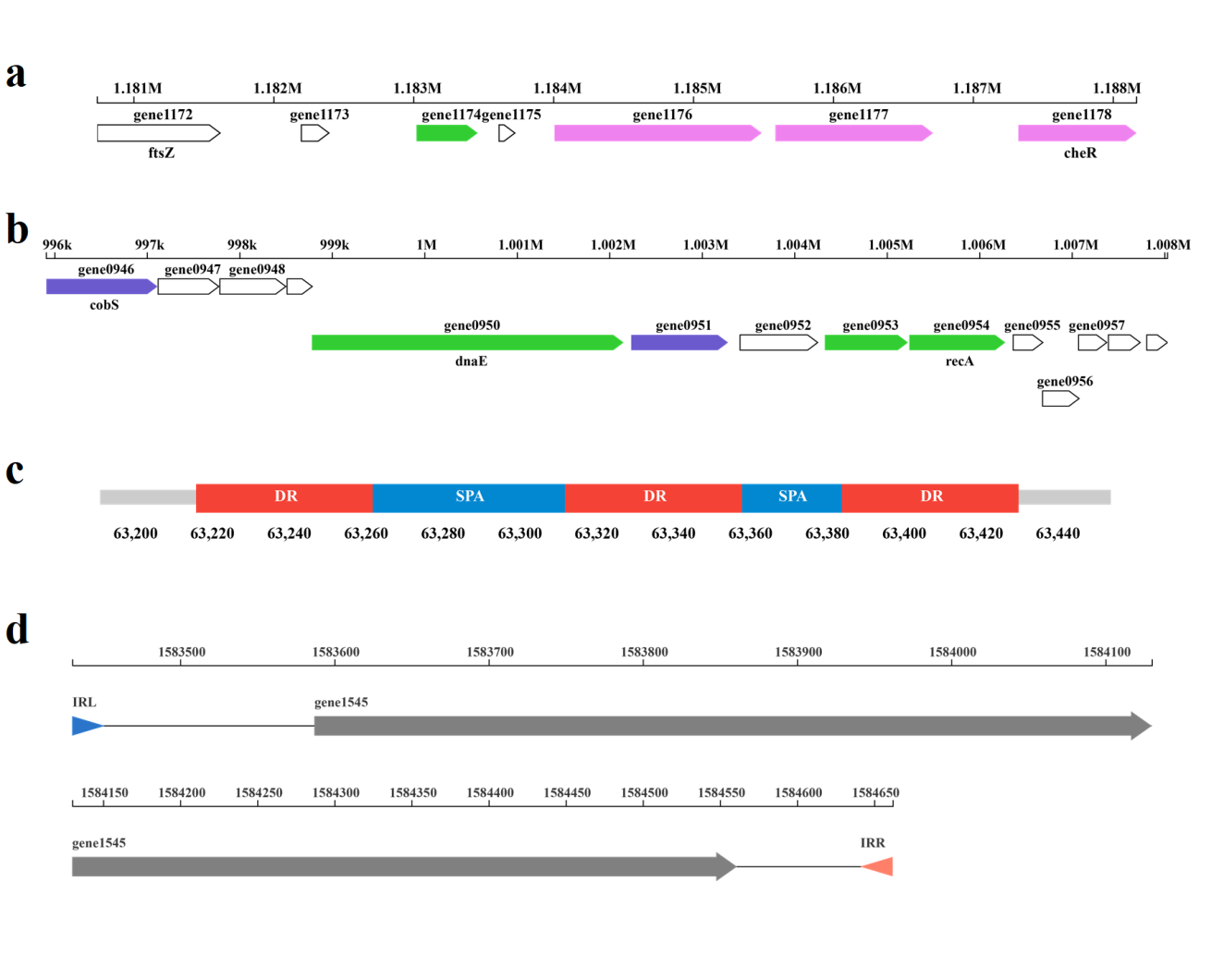


**Fig. S2.** Linear or structural diagrams of MGEs. **a**. Linear map of gene islands; **b**. Linear map of prophages; **c**. Predicted structure of the CRISPR/Cas systems; **d**. The structure of the inserted sequence.


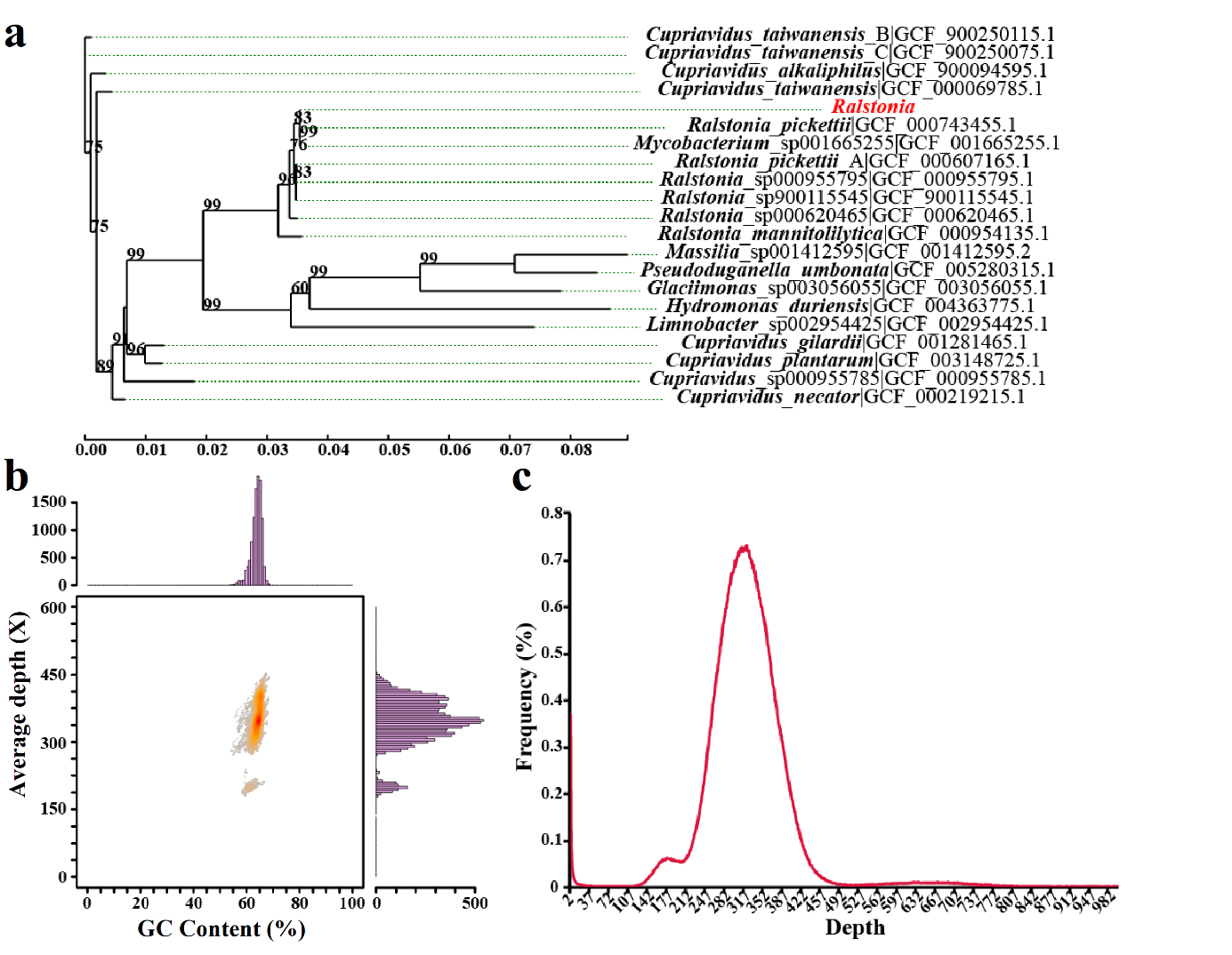


**Fig. S3.** Phylogenetic tree and genome assessment. **a**. Phylogenetic tree of high-level polymyxin-resistant MDRB; **b**. GC_depth distribution; **c**. K-mer frequency distribution.


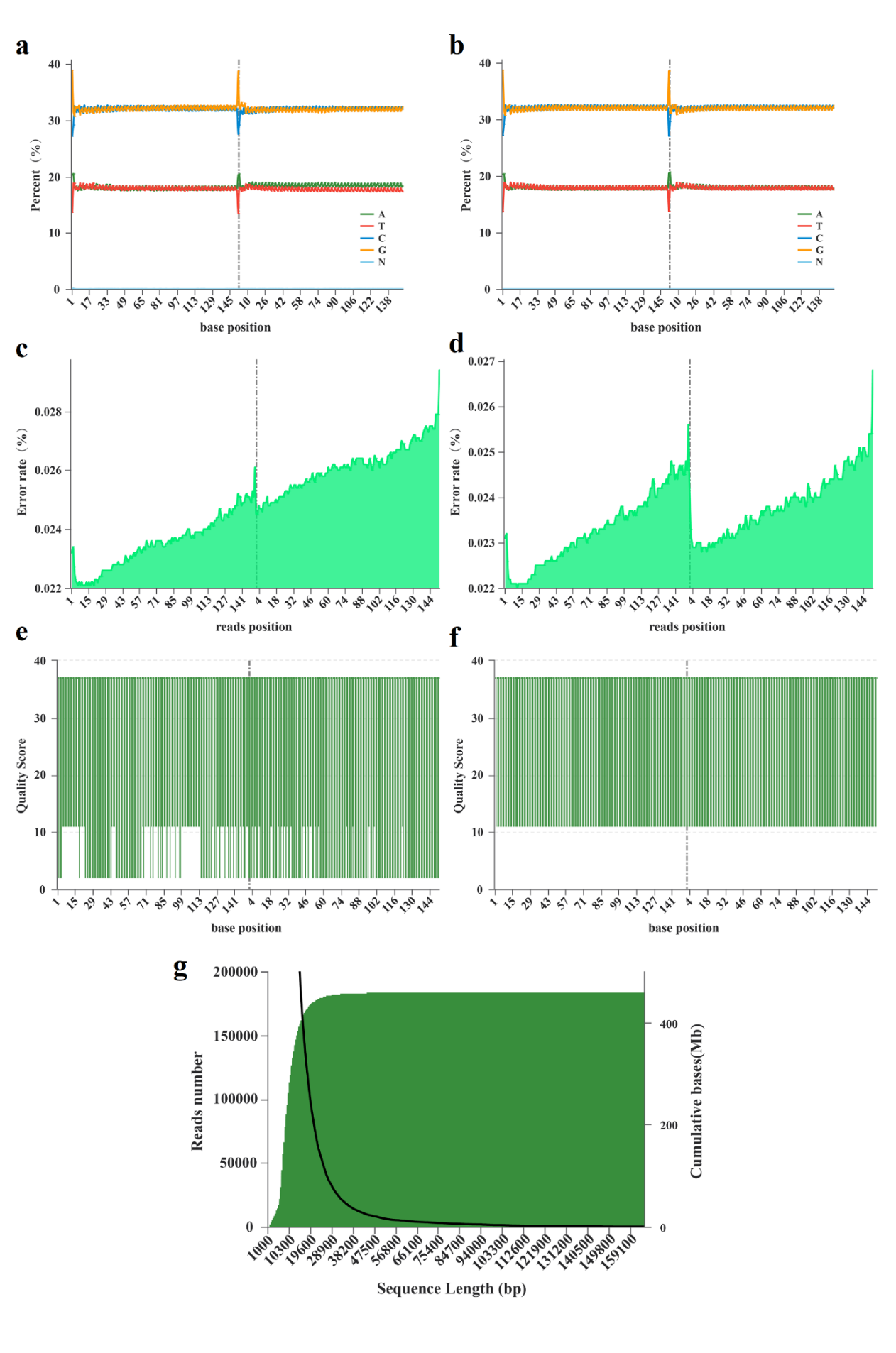


**Fig. S4.** Genome sequencing data quality control. **a**. Raw Reads base composition distribution; **b**. Clean Reads base composition distribution; **c**. Raw Reads base error rate distribution; **d**. Clean Reads base error rate distribution; **e**. Raw Reads base quality distribution; **f**. Clean Reads base quality distribution; **g**. Clean Reads length distribution.


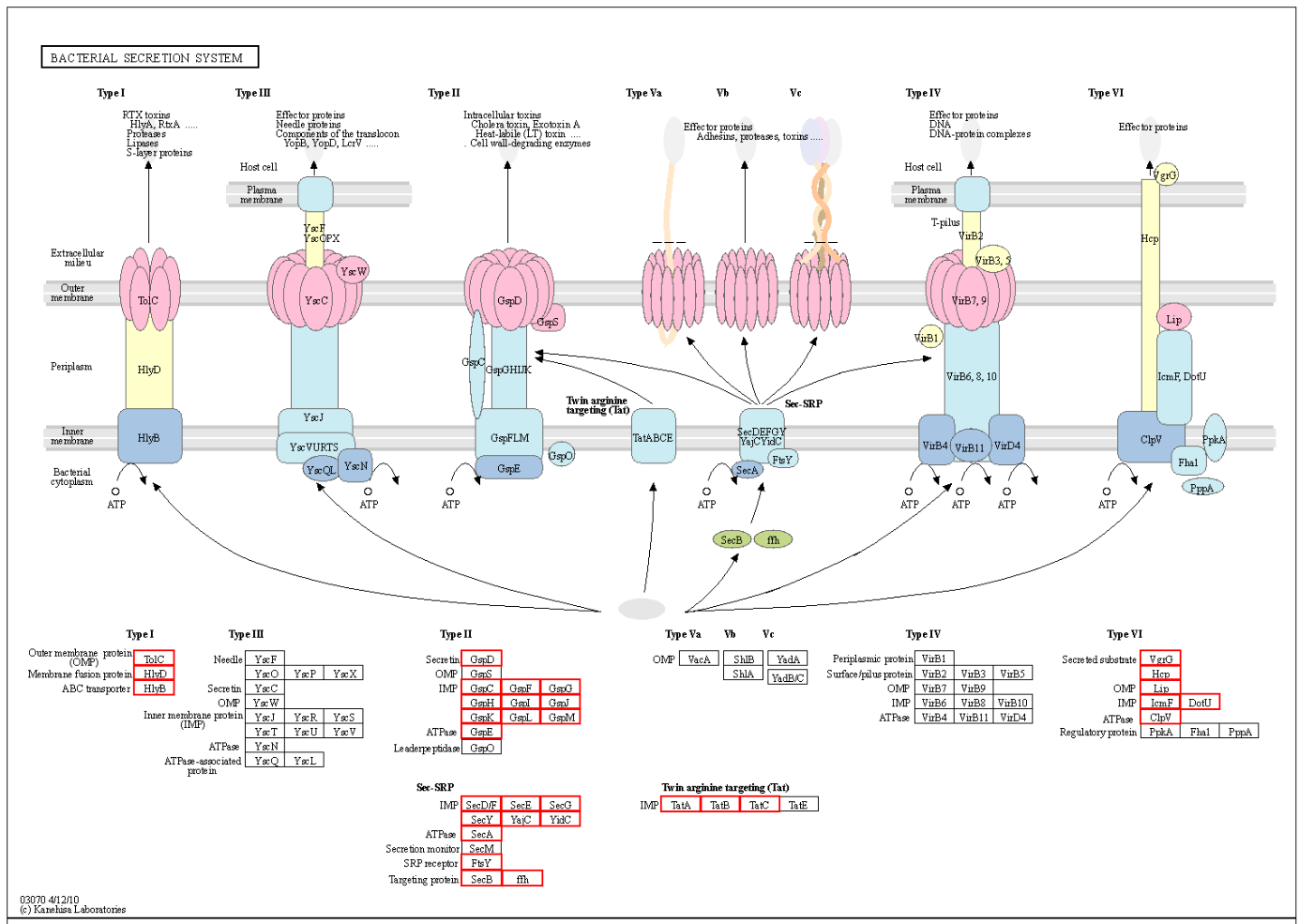


**Fig. S5.** Secretory system pathway.


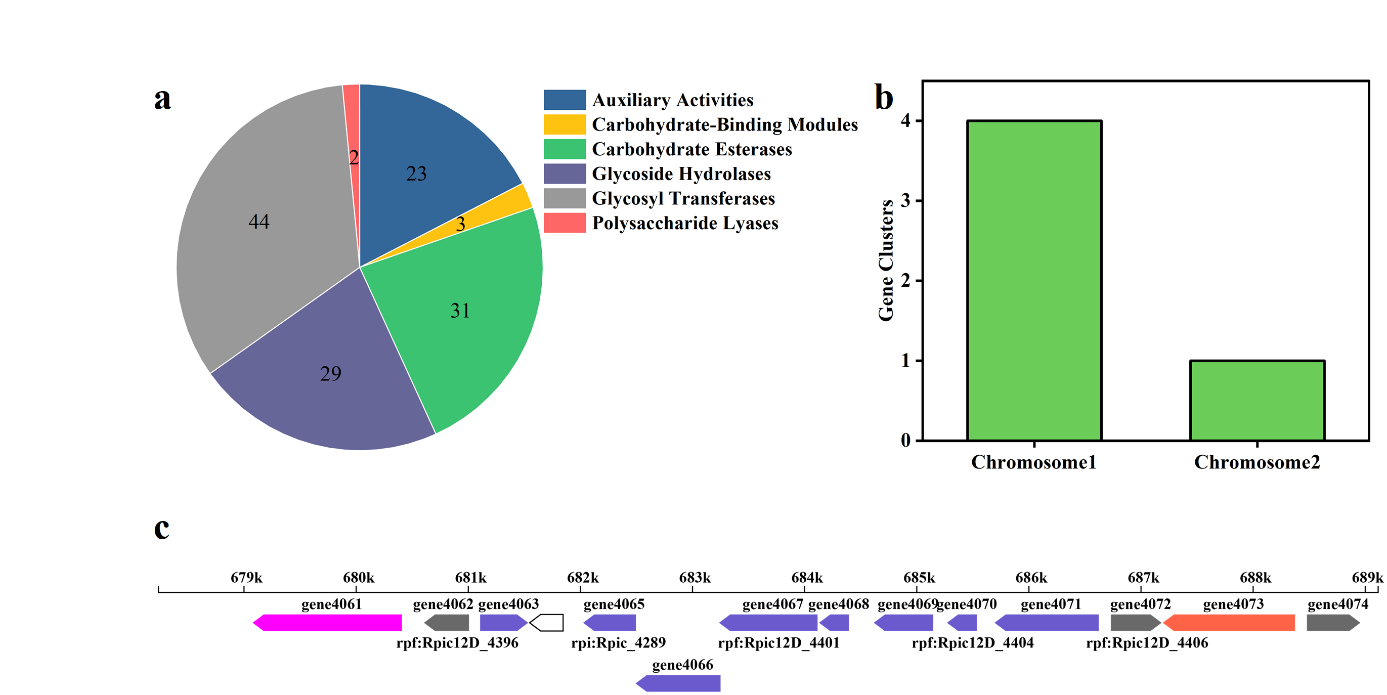


**Fig. S6.** Carbohydrate-active enzyme and secondary metabolite synthesis gene clusters. **a**. Carbohydrate activity enzyme annotation graph; **b**. Secondary metabolite synthesis gene cluster analysis statistics; **c**. Linear map of secondary metabolite synthesis gene clusters.

**References**

1. Liang, Z., Yu, Y., Ye, Z., Li, G., Wang, W.,An, T. Pollution profiles of antibiotic resistance genes associated with airborne opportunistic pathogens from typical area, Pearl River Estuary and their exposure risk to human. Environment International. 2020; 143: 105934.

2. Chen, X., Yin, H., Li, G., Wang, W., Wong, P.K., Zhao, H. *et al.* Antibiotic-resistance gene transfer in antibiotic-resistance bacteria under different light irradiation: Implications from oxidative stress and gene expression. Water Research. 2019; 149: 282-291.

3. Liu, Y., Cai, Y., Li, G., Wang, W., Wong, P.K.,An, T. Response mechanisms of different antibiotic-resistant bacteria with different resistance action targets to the stress from photocatalytic oxidation. Water Research. 2022; 218: 118407.

4. Nie, X., Li, G.Y., Gao, M.H., Sun, H.W., Liu, X.L., Zhao, H.J. *et al.* Comparative study on the photoelectrocatalytic inactivation of Escherichia coli K-12 and its mutant Escherichia coli BW25113 using TiO2 nanotubes as a photoanode. Applied Catalysis B: Environmental. 2014; 147: 562-570.

5. Laffite, A., Al Salah, D.M.M., Slaveykova, V.I., Otamonga, J.P.,Pote, J. Impact of anthropogenic activities on the occurrence and distribution of toxic metals, extending-spectra beta-lactamases and carbapenem resistance in sub-Saharan African urban rivers. Science of the Total Environment. 2020; 727: 138129.

6. Subirats, J., Di Cesare, A., Varela Della Giustina, S., Fiorentino, A., Eckert, E.M., Rodriguez-Mozaz, S. *et al.* High-quality treated wastewater causes remarkable changes in natural microbial communities and intI1 gene abundance. Water Research. 2019; 167: 114895.

7. Guo, M.T.,Zhang, G.S. Graphene oxide in the water environment could affect tetracycline-antibiotic resistance. Chemosphere. 2017; 183: 197-203.

8. Sousa, J.M., Macedo, G., Pedrosa, M., Becerra-Castro, C., Castro-Silva, S., Pereira, M.F.R. *et al.* Ozonation and UV254nm radiation for the removal of microorganisms and antibiotic resistance genes from urban wastewater. Journal of Hazardous Materials. 2017; 323: 434-441.

9. Tong, J., Lu, X., Zhang, J., Sui, Q., Wang, R., Chen, M. *et al.* Occurrence of antibiotic resistance genes and mobile genetic elements in enterococci and genomic DNA during anaerobic digestion of pharmaceutical waste sludge with different pretreatments. Bioresource Technology. 2017; 235: 316-324.

10. Ferro, G., Guarino, F., Cicatelli, A.,Rizzo, L. beta-lactams resistance gene quantification in an antibiotic resistant Escherichia coli water suspension treated by advanced oxidation with UV/H2O2. Journal of Hazardous Materials. 2017; 323: 426-433.

11. Kayali, O.,Icgen, B. Untreated HWWs emerged as hotpots for ARGs. Bulletin of Environmental Contamination and Toxicology 2020; 104: 386-392.

12. Augsburger, N., Mantilla-Calderon, D., Daffonchio, D.,Hong, P.Y. Acquisition of extracellular DNA by Acinetobacter baylyi ADP1 in response to solar and UV-C254nm disinfection. Environmental Science & Technology. 2019; 53: 10312-10319.

13. Zhang, Y., Zhuang, Y., Geng, J., Ren, H., Xu, K.,Ding, L. Reduction of antibiotic resistance genes in municipal wastewater effluent by advanced oxidation processes. Science of the Total Environment. 2016; 550: 184-191.

14. Mao, D., Yu, S., Rysz, M., Luo, Y., Yang, F., Li, F. *et al.* Prevalence and proliferation of antibiotic resistance genes in two municipal wastewater treatment plants. Water Research. 2015; 85: 458-466.
